# Microparticle-mediated CRISPR DNA delivery for genome editing in poplar

**DOI:** 10.3389/fpls.2023.1286663

**Published:** 2023-11-13

**Authors:** Lennart Hoengenaert, Jan Van Doorsselaere, Ruben Vanholme, Wout Boerjan

**Affiliations:** ^1^ Department of Plant Biotechnology and Bioinformatics, Ghent University, Ghent, Belgium; ^2^ Center for Plant Systems Biology, VIB, Ghent, Belgium; ^3^ VIVES, University of Applied Sciences, Roeselare, Belgium

**Keywords:** CRISPR, gene editing, particle bombardment, poplar, targeted insertions, tissue culture, transgene-free

## Abstract

The use of CRISPR/Cas9 is currently the method of choice for precise genome engineering in plants, including in the biomass crop poplar. The most commonly used method for delivering CRISPR/Cas9 and its components in poplar is via *Agrobacterium-*mediated transformation, that besides the desired gene-editing event also results in stable T-DNA integration. Here we explore the delivery of the gene-editing reagents via DNA-coated microparticle bombardment into the model tree *Populus tremula x P. alba* to evaluate its potential for developing transgene-free, gene-edited trees, as well as its potential for integrating donor DNA at specific target sites. Using an optimized transformation method, which favors the regeneration of plants that transiently express the genes on the delivered donor DNA, we regenerated gene-edited plants that are free of the *Cas9* and the antibiotic resistance-encoding transgenes. In addition, we report the frequent integration of donor DNA fragments at the Cas9-induced double-strand break, opening opportunities toward targeted gene insertions.

## 1 Introduction

The production of pulp, bio-based materials and energy at an industrial scale benefits from plantation forestry with highly productive tree species. Preferably, such trees have excellent nutrient uptake abilities, exhibit resilience against climate change, and are equipped to withstand both biotic and abiotic stresses. The development of such trees requires the smart combination of conventional and new breeding technologies. The use of the clustered regularly interspaced palindromic repeats (CRISPR)/CRISPR-associated protein 9 (Cas9) system is the method of choice for precise genome engineering in plants, including the biomass crop poplar (Fan et al., 2015; Zhou et al., 2015; Anders et al., 2023; Sulis et al., 2023). In trees, CRISPR gene-editing reagents, i.e., the guide RNA (gRNA) and Cas9 protein, are typically delivered via *Agrobacterium tumefaciens-*mediated transformation, often resulting in the stable genomic integration and expression of the transfer DNA (T-DNA). However, the presence of active Cas9-expression cassettes in the genome of the plant impedes their valorization, as the current regulatory framework complicates field testing and commercialization of plants harboring gene-editing reagents. In addition, the introgression of CRISPR-cassettes into wild relatives may be seen as a potential risk factor, as the dominant editing by the Cas9-nuclease and the gRNA might alter the frequency of the edited allele in natural populations. In sexually propagated plants, T-DNAs can be eliminated by Mendelian segregation, yielding transgene-free plants. However, plants that are asexually propagated and/or are highly heterozygous, e.g., poplar, grape vine, sugarcane, etc., lose their genetic constitution upon sexual reproduction. To overcome these limitations, several groups have tried to transiently express the gene-editing machinery in crops via *Agrobacterium-*mediated transformation. After all, multiple studies in Arabidopsis demonstrate the transfer and subsequent expression of a T-DNA, without integration into the plant genome when no selective pressure is applied (De Buck et al., 1998; De Buck et al., 2000; Ghedira et al., 2013). Indeed, transient expression of a Cas9-coding DNA followed by successful gene editing was reported in tomato, potato, apple, and pear tree (Jacobs et al., 2017; Chen et al., 2018; Charrier et al., 2019; Danilo et al., 2019; Veillet et al., 2019; Banfalvi et al., 2020). However, these studies showed relatively low gene-editing efficiencies and therefore rely on labor-intensive screening protocols to identify gene-edited shoots that are free of transgenes.

Alternative strategies for the delivery of gene-editing reagents into plant cells involve either the delivery of ribonucleoproteins (RNPs) or the transient expression of a gRNA- and Cas9-coding DNA/RNA in protoplasts or callus tissue. Although protoplast cultures allow the delivery of gene-editing reagents to the majority of cells (Lin et al., 2018), the culture and subsequent regeneration remains tedious and is currently limited to a handful of species (Woo et al., 2015; Malnoy et al., 2016; Andersson et al., 2017; Lin et al., 2018; Murovec et al., 2018; Choi et al., 2021). Moreover, this approach is known to induce somaclonal variation, i.e., undesired mutations, and large genome rearrangements, potentially leading to an altered phenotype (Serres et al., 1991; Fossi et al., 2019). Another commonly used strategy utilizes mechanical force to deliver microparticles coated with gene-editing reagents into a tissue of interest. As such, Cas9-coding DNA and RNA have been delivered into wheat embryos, yielding gene-edited plantlets free of Cas9-coding DNA upon regeneration on non-selective medium (Zhang et al., 2016). Similarly, Cas9-free, gene-edited wheat and maize could be obtained via particle gun-mediated delivery of RNPs (Svitashev et al., 2016; Liang et al., 2017; Poddar et al., 2023). Microparticle-mediated DNA delivery methods have also been used previously for the transient expression of foreign genes in tree species, including in poplar (Devantier et al., 1993; Nowak et al., 2004; Canto, 2016). A frequently reported downside of this method is its tendency to cause double-strand breaks (DSBs) due to the high force used to deliver microparticles, which can lead to the integration of delivered donor DNA and/or chromosomal rearrangements (Liu et al., 2019; Yue et al., 2022).

In this study, we explore the delivery of CRISPR DNA via microparticles in poplar to assess its potential for developing transgene-free, gene-edited trees, as well as its potential for the on-target integration of donor DNA. As a target gene to evaluate proof-of-concept gene editing, we selected the *CINNAMOYL-CoA REDUCTASE 2* (*CCR2*) gene from poplar, encoding a key enzyme involved in lignin biosynthesis (Leplé et al., 2007; Van Acker et al., 2014; Vanholme et al., 2019; De Meester et al., 2020). Plants with biallelic mutations in the *CCR2*-encoding sequence are easily distinguished from their wild-type (WT) controls because of their stunted growth and dark-green coloration of the leaves, phenotypes that are already visible at the tissue-culture stage (Leplé et al., 2007; De Meester et al., 2020).

## 2 Materials and methods

### 2.1 Plant material and callus induction

Poplar (*Populus tremula x P. alba*, INRA-clone 717-1B4) shoot cultures were grown *in vitro* on ½ Murashige and Skoog medium supplemented with 0.5 mg/L indole-3-acetic acid (IAA) (medium M1/2WT, **Supplementary Table S1**) (Leplé et al., 1992). Plantlets were grown and maintained on this medium in a tissue culture room (16-h photoperiod with a light intensity of ± 70 µmol m^-2^ s^-1^, 70% humidity, 21°C). Four- to six-week-old plants were used for callus induction. Internodes of 0.5 cm to 1.0 cm in length were excised and wounded with a scalpel blade before being cultured for 17 days in the dark on callus-inducing medium (medium M1, **Supplementary Table S1**) (Leplé et al., 1992).

### 2.2 Transformation vectors

To validate poplar callus transformation, a 4.6-kb *pKAR6-GFP* plasmid was used that encodes a codon-optimized, ER-targeted eGFP protein (Robert Blanvillain, unpublished data) (Thomson et al., 2011) (**Supplementary Figure S1**). Gene expression was driven by the *CaMV-35S* promoter and terminated by a *CaMV-35S* terminator. For genome-editing purposes, the *p201NCas9:gRNA_CCR2* vector was used (De Meester et al., 2020), here named as the *pCCR2-CRISPR* plasmid. In summary, the vector contains the *CCR2* gRNA sequence driven by the *Medicago truncatula U6* promoter and terminated by *T6*. *Cas9*-expression is driven by the *CaMV-35S* promoter with a double *35S* enhancer and terminated by the *NOS* terminator. In addition, the vector contains the *NPTII* gene, encoding *NEOMYCIN PHOSPHOTRANSFERASE II*, conferring resistance to kanamycin sulphate. All vectors were upscaled in *E. coli* and purified using either a Macherey-Nagel NucleoBond Maxi kit or a Thermo Scientific GeneJET Plasmid Miniprep kit, according to manufacturing procedures.

### 2.3 Biolistic transformation and transformation validation

Gold microparticles (3 mg/50 µL H_2_O; BioRad Gold 0.6-µm diameter) were coated by addition of 50 µL 2.5 M CaCl_2_, 3 µg vector DNA (1 µg/µL) and 1.7 µL 0.1 M spermidine under continuous vortexing. The pellet was washed in 250 µL 100% ethanol and subsequently resolubilized in 50 µL 100% ethanol. Prior to transformation, approximately 20 calli were centered on a Petri dish containing callus-inducing medium (M1, **Supplementary Table S1**). Biolistic transformation was performed using the Particle Delivery System PDS-1000/He (BioRad, Munich, Germany) using a vacuum of 28 inch Hg, a helium pressure of 1100 PSI and a target distance of 6 cm. Immediately after bombardment, calli were stored in the dark for 24-h, allowing them to recover from the bombardment. Next, calli to which *pKAR6-GFP-*coated microparticles were delivered were visually screened using a fluorescence stereomicroscope (Leica M165 FC microscope with a Leica DFC7000 T camera, Wetzlar, Germany). After ten days, fluorescence levels were reevaluated using the same fluorescence stereomicroscope.

### 2.4 Selection and plant regeneration

To enrich for transformed cells, 24 h after the biolistic delivery of gene-editing reagents, all calli were transferred to shoot induction medium supplemented with kanamycin sulfate (M3K, **Supplementary Table S1**), upon which they were cultivated in the dark for 48 h at 37°C. Subsequent to the selective treatment, calli were recovered to non-selective M3 medium (**Supplementary Table S1**) (Leplé et al., 1992), and grown in a tissue culture room (16-h photoperiod with a light intensity of ± 70 µmol m^-2^ s^-1^, 70% humidity, 21°C). After 6 weeks of culture, green spherical cell clusters appeared on white-to-yellowish parental calli. All individual cell clusters were separated from the parental calli and cultivated on fresh M3 medium. The parental callus was discarded. Cultivation of cell clusters continued for 7 months, during which multiple shoots were harvested. On a monthly basis, the M3 medium was refreshed. Harvested shoots were transferred to root induction medium (M1/2WT, **Supplementary Table S1**). A selection (**Supplementary Table S2**) of the gene-edited population was maintained on this M1/2WT medium through cycles of vegetative propagation (approximately 1 cycle every 5-6 months).

### 2.5 Molecular analyses

Genomic DNA was isolated from green cell clusters using the Edwards DNA extraction method (Edwards et al., 1991). *PtaCCR2* amplicons were obtained by PCR using the following primers and settings: 5x Q5 reaction buffer (NEB), 10 mM dNTPs (Promega), 500 units Q5 high fidelity DNA-polymerase (NEB), 10 μM *PtaCCR2* forward primer (5´-TACAYGGTAATTAATGGTGG-3´), 10 μM *PtaCCR2* reverse primer (5´-GATACCTTGGTGTTCTTGC-3´) and 2 μL DNA template in MQ-H_2_O. PCR was carried out with an initial denaturation step at 98°C for 30 s followed by 35 cycles consisting of steps of 98°C for 10 s and 55°C for 20 s and 72°C for 8 s. Allele-specific amplicons were obtained using the *PtaCCR2_tremula_F* (5´-CTCACACAGCTTATTTTAGCACAC-3´), *PtaCCR2_tremula_R* (5´-TTGGTTCAGGTGTTAGCTTGG-3´), *PtaCCR2_alba_F* (5´-CTCACACAGCTAATTTTAGCACAA-3´), and *PtaCCR2_alba_R* (5´-TGACTGGTCCACAAGAAGGA-3´) primers. The presence of DNA inserts as in category II and III mutations was evaluated via PCR with primer extension at 72°C for up to 1 min, to allow amplification of longer PCR fragments. The PCR was terminated with a final extension step of 2 min at 72°C and ultimately cooled to 4°C. The presence of the PCR amplicon was checked on a 1.2% agarose gel (100V) with a molecular weight marker (Promega, Benchtop 1-kb ladder). Prior to sequence analysis, *PtaCCR2* amplicons were purified via HighPrep™ (MAGBIO AC-60050) post-PCR clean-up. Purified *CCR2* amplicons were sent for Sanger sequencing (Eurofins Genomics, Germany) and the obtained sequencing chromatograms were analyzed to detect significant gene editing using TIDE (Brinkman et al., 2014), ICE (Synthego Performance Analysis, ICE Analysis. 2019. v2.0. Synthego; October 2020) (Hsiau et al., 2018), and Geneious Prime 2022.2.1.

PCR-based genotyping to detect the presence of DNA encoding *Cas9*, *NPTII* and *CCR2-gRNA* was performed using a gene-specific forward (*Cas9*: 5´-CTGAGTTGGATAAAGCCGGC-3´; *NPTII*: 5´-CCGCTCAGAAGAACTCGTCAA-3´; *CCR2-gRNA*: 5´-TATGTCCTGATAGCGGTCCG-3´) and reverse (*Cas9*: 5´- TTGGCCAGTGTAATCTCGGT-3´; *NPTII*: 5´-GTGCTCGACGTTGTCACTGAA-3´; *CCR2-gRNA*: 5´- AGCAGTGCACCTTGTCTTTG-3´) primer. The PCR was carried out using GoTaq® G2 (Promega) with an initial denaturation step at 95°C for 3 min, followed by 35 cycles consisting of steps of 95°C for 30 s, 55°C for 30 s, and 72°C for 45 s. The PCR was terminated with a final extension step of 2 min at 72°C and ultimately cooled to 4°C. PCR amplicons were separated in a 1.2% agarose gel at 100V for 30 min.

### 2.6 Modeling of the CCR2 protein structure

Allele-specific transcript sequences of *CCR2* were obtained from the AspenDB (Zhou et al., 2015). The most abundant transcript was retained and translated into its corresponding protein sequence. Additionally, the sequence was manually edited to introduce the mutation observed in line 116.2, resulting in the deletion of Ile-114. The CCR2 protein sequence of the haploinsufficient allele in CCR2(-/*) line 12 was obtained from De Meester et al. (2020). Three-dimensional protein structures of WT and both mutated CCR2 proteins were modeled using the crystal structure of the *Petunia hybrida* CCR protein [PDB ID: 4R1S, (Pan et al., 2014)] as a modeling template in the fully automated protein structure homology-modeling server SWISS-MODEL (Biasini et al., 2014). Protein models were visualized using PyMOL (**Supplementary Figure S2**).

## 3 Results

### 3.1 Validation of the microparticle-mediated transformation of *Populus tremula* x *Populus alba*

**Figure 1:**
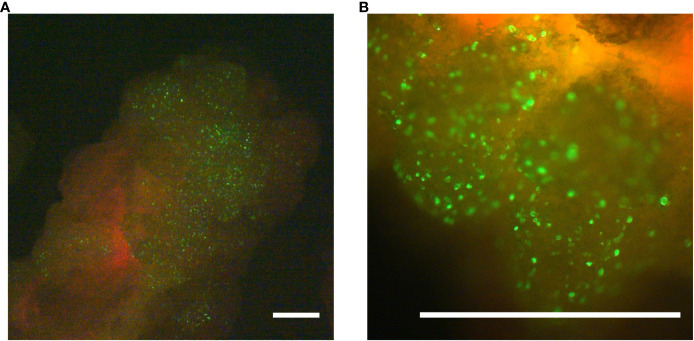
Transformation validation using microprojectile-based delivery of an ER-localized GFP-protein. Twenty-four hours after the biolistic delivery of the *pKAR6-GFP* reporter plasmid into *P. tremula x P. alba* callus, expression was evaluated using a fluorescence stereomicroscope. Scale bars represent 1 mm. **(B)** is a close-up of the same callus shown in **(A)**.

The poplar hybrid *Populus tremula x P. alba* (*Pta*) clone 717-1B4 is a frequently used model for molecular genetic research in trees. To evaluate whether undifferentiated callus from this poplar hybrid allows the expression of transgenes delivered via microparticles, a GFP-reporter plasmid (*pKAR6-GFP*), encoding an ER-targeted, codon-optimized eGFP, was delivered into 17-day-old callus. Twenty-four hours after the delivery of *pKAR6-GFP-*coated microparticles, bombarded samples were analyzed by fluorescence microscopy. At this stage, observed fluorescent signals originated from cells that actively expressed the reporter plasmid, regardless whether or not the reporter plasmid had integrated into the plant genome. Fluorescence was observed from multiple discrete cells at the surface of the callus tissue (**Figure 1**). To evaluate the extent of transient expression, fluorescence was reevaluated on the same calli ten days after bombardment. The bulk of the fluorescent signal had disappeared, suggesting that the majority of delivered marker DNA was only transiently expressed in poplar callus.

### 3.2 Biolistic delivery of gene-editing reagents into poplar callus

Two strategies were designed to investigate the efficiency of gene editing while avoiding stable integration of transgenes into the poplar genome. First, we investigated whether gene-edited shoots could be regenerated without the use of selective medium (Strategy I). Therefore, a *pCCR2-CRISPR* plasmid that was previously successfully used for *Agrobacterium*-mediated transformation and that encodes a gRNA targeting the *CCR2* gene of poplar and a Cas9 nuclease, was coated onto gold microparticles and delivered into 17-day-old poplar callus. Following biolistic transformation, bombarded calli were kept on non-selective shoot induction medium to allow the regeneration of shoots. Over a period of six months, 363 shoots were harvested from the calli and transferred to root-inducing medium. Subsequently, all shoots were visually screened for the characteristic biomass yield penalty and dark-green coloration of the leaves that is typically observed for biallelic *ccr2-*knock-out lines (De Meester et al., 2020). However, none of the 363 regenerated plantlets showed a *ccr2*-knock-out phenotype, implying that the used methodology failed to generate shoots with biallelic knock-out mutations in *CCR2*, and, consequently, that it would be hard to generate transgene-free, gene-edited poplars via this methodology.

**Figure 2:**
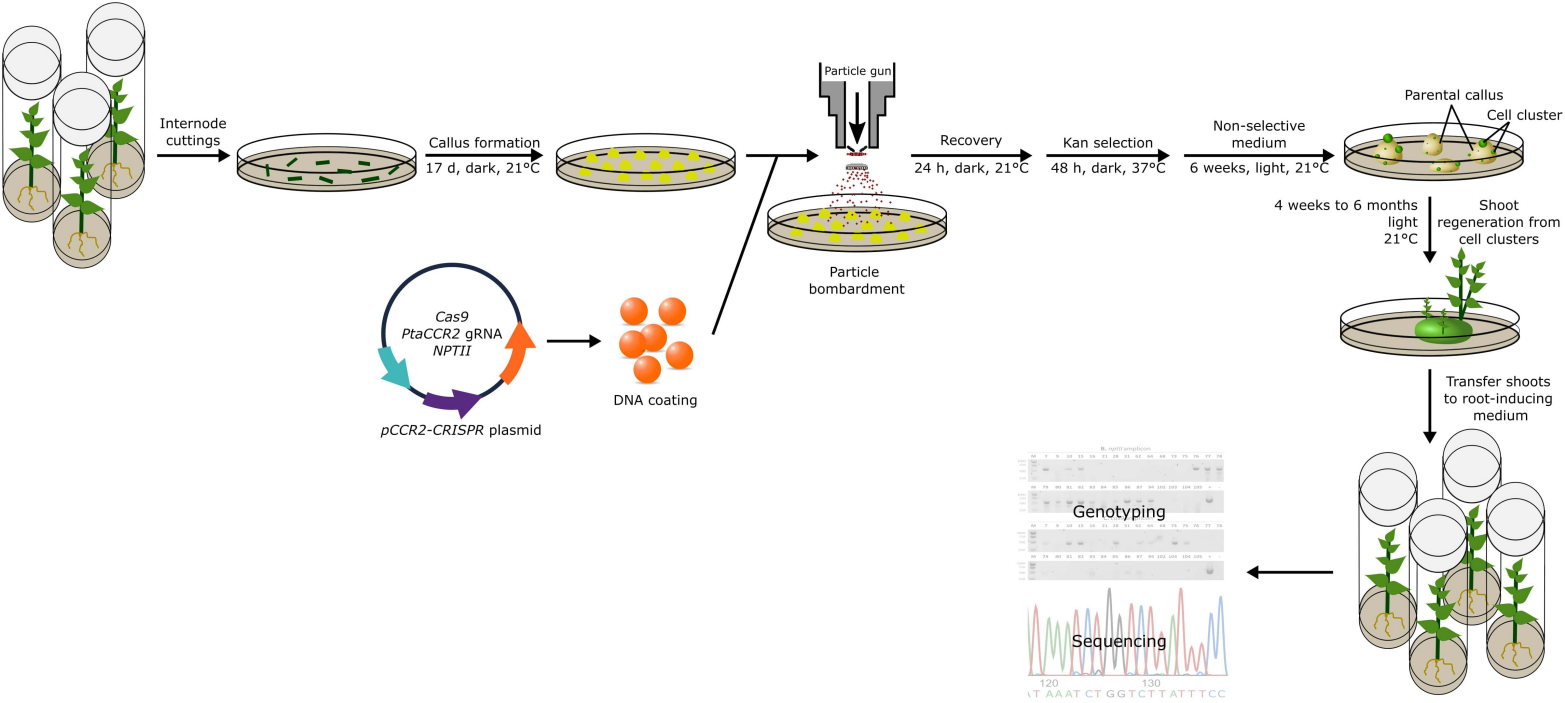
Strategy to obtain genome-edited poplar. Schematic representation of the transformation method and analyses pipeline described in this study. In summary, the *pCCR2-CRISPR* plasmid was coated onto gold microparticles and delivered into 17-day-old undifferentiated poplar callus tissue. After 24 h of recovery, the calli were subjected to a 48-h selective treatment at elevated temperature before being transferred to non-selective medium at room temperature. After 6 weeks, green spherical cell clusters developed on the white-to-yellowish parental calli. Individual cell clusters were further cultivated and used for sequencing analyses or the regeneration of plantlets. The first shoots were harvested after 4 weeks and continued over a period of 6 months. Through genotyping and sequence analyses, *CCR2* gene-edited shoots were identified.

**Figure 3:**
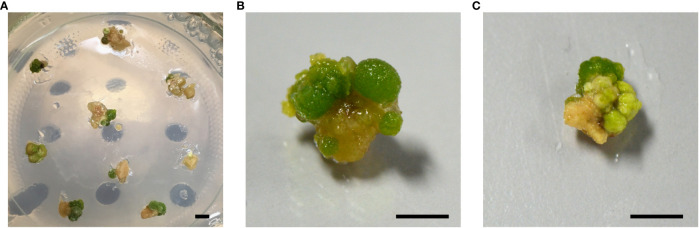
Formation of green cell clusters. After 6 weeks of recovery on non-selective medium, green spherical cell clusters developed on white-to-yellowish parental calli. A single parental callus can develop one or multiple green cell clusters. Independent cell clusters were isolated and either sacrificed in a fast genomic screening method for a potentially edited *CCR2* gene, or cultured for the regeneration of shoots. **(A)** Overview of multiple parental calli developing one or more green cell clusters. **(B, C)** Close-up of parental calli developing multiple green cell clusters. Scale bars represent 5 mm.

To enhance the regeneration of transgene-free, gene-edited poplars, we explored a second strategy. We argued that a short selective treatment could possibly enrich cells that transiently express the genes encoded by the delivered DNA (Strategy II). To this end, we took advantage of the *NPTII* selectable marker that was encoded by the *pCCR2-CRISPR* plasmid to allow for a 48-h temporary selection in favor of cells that actively express the genes on the delivered DNA. In addition, a concurrent treatment at 37°C was included, as the use of a heat pulse was shown to promote the Cas9-editing efficiency (Leblanc et al., 2018; Blomme et al., 2022). Following the temporary selective treatment on kanamycin-containing medium, all calli were transferred to non-selective medium for shoot induction (**Figure 2**). After 6 weeks on non-selective medium, green spherical cell clusters developed (**Figure 3**) on white-to-yellowish parental calli, which did not show any signs of growth or division. Of 300 calli subjected to biolistics and the subsequent selective treatment, 148 (49.3%) developed one or more of such proliferating green cell clusters. We hypothesized that these cell clusters originated from cells that obtained the *pCCR2-CRISPR* plasmid and therefore survived the temporary selective treatment. After all, previous experiments demonstrated that calli which did not receive a selective treatment with kanamycin turned immediately green and readily developed shoots that were phenotypically indistinguishable from WT shoots.

To determine whether genome editing occurred at the *CCR2* locus, white-to-yellowish parental calli that developed green cell clusters were divided into two groups. One group (58 calli) was used for DNA sequence analysis (a process that is destructive for the calli, preventing their further regeneration into shoots), while the second group (90 calli) was further evaluated for shoot regeneration.

### 3.3 Evaluating *pCCR2-CRISPR* delivery in green proliferating cell clusters

**Figure 4:**
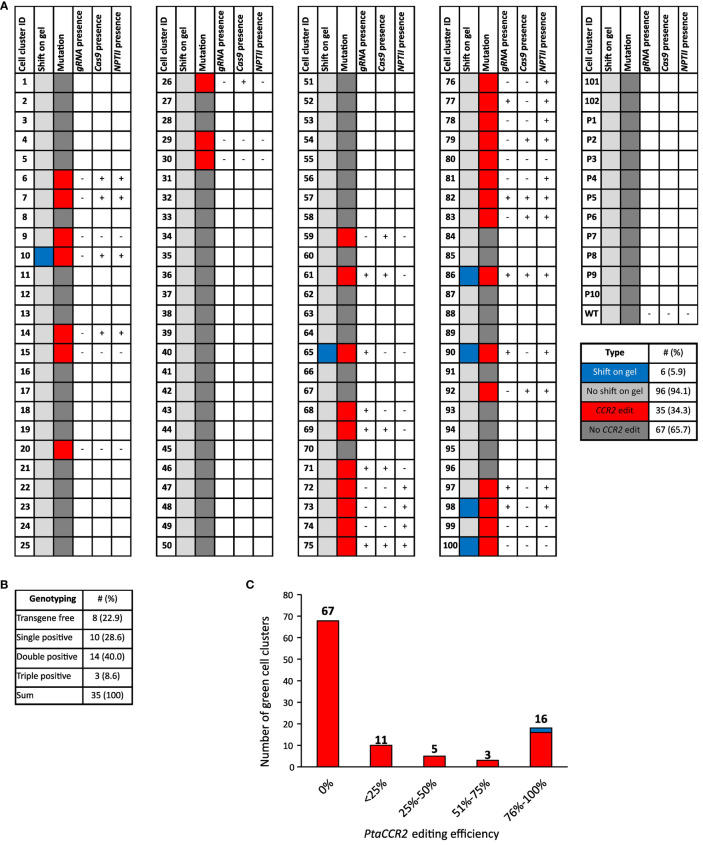
Schematic overview of the cell cluster population together with sequencing and genotyping results. **(A)** Genomic DNA was prepared from 102 green proliferating cell clusters (cell cluster ID 1-102) and 10 randomly selected white-to-yellowish parental calli (P1-P10). WT represents callus that was not subjected to biolistic DNA delivery. Genomic DNA was analyzed for edits in the *CCR2* gene through amplicon size determination via PCR followed by gel electrophoresis and trough PCR followed by sequencing. The results for the amplicon size are shown in the column ‘shift on gel’. Cell clusters of which the *CCR2* amplicon was larger than that of the WT upon gel electrophoresis and those of the same size as WT, are indicated in blue and light gray, respectively. The *CCR2* sequencing results are shown in the column ‘mutations’. Cell clusters with significant *CCR2* editing are depicted in red, while cell clusters without significant edits are depicted in dark gray. Cell clusters that contained edits in the *CCR2* gene were further analyzed for the presence of the *pCCR2-CRISPR* plasmid. Therefore, genomic DNA was tested for the presence of *Cas9*, *CCR2*-*gRNA* and the *NPTII* selectable marker gene via PCR-based genotyping. Samples that tested positive for either *Cas9*, *gRNA* or the *NPTII* selectable marker gene are indicated by +, cell clusters for which none of the three PCR products could be detected are indicated by -. **(B)** Summary of the PCR-based genotyping for *Cas9*, *gRNA* or the *NPTII* selectable marker gene. **(C)** Distribution of the *CCR2*-editing efficiency in cell clusters upon microparticle-mediated delivery of the *pCCR2-CRISPR* plasmid. Gene-editing efficiency varied between independent cell clusters, ranging from biallelic edits to chimeric cell clusters and clusters with low editing efficiency. Note: chimeric cell clusters can arise from gene-editing activity after cell division or from the non-exclusive manual separation of cell clusters from other cell clusters or their parental callus. For two cell clusters (10 and 86, represented in blue), no conclusive sequencing profile could be obtained. Their *CCR2* editing was deduced from the shift in their respective *CCR2* amplicons on the agarose gel.

Genomic DNA extracts were prepared from 102 green proliferating cell clusters originating from 58 parental calli. Prior to sequence analysis of the part of the *CCR2* gene covering the Cas9 target site, its amplicon was visualized through gel electrophoresis. Interestingly, *CCR2* amplicons of six (5.9%) cell clusters had a larger size compared to the wild-type *CCR2* amplicon, suggesting the insertion of a stretch of nucleotides into the target locus (**Figure 4A**, **Supplementary Figure S3**). Sanger sequencing of the PCR-amplicons indicated significant editing at the *CCR2* locus in 35 (34.3%) of the analyzed cell clusters (**Figure 4A**). Gene editing varied among independent events ranging from biallelic edits to chimeric cell clusters and clusters with low editing efficiencies (**Figure 4C**, **Supplementary Table S3**). Notably, no edits were observed in ten randomly selected white-yellowish parental calli, suggesting that these regions were most likely not transformed, or did not express the *pCCR2-CRISPR* plasmid, and consequentially suffered from the temporary kanamycin selection (**Supplementary Table S4**).

Next, we tested whether the cell clusters that were either fully or partially edited had a stable T-DNA insertion. We therefore checked for the presence of *Cas9*, *CCR2-gRNA* and the *NPTII* selectable marker genes in the extracted DNA. Eight out of thirty-five (22.9%) gene-edited cell clusters were free of the three tested transgenes and could thus potentially be T-DNA-free (**Figure 4B**, **Supplementary Figure S4**).

### 3.4 Evaluating *pCCR2-CRISPR* delivery in regenerated plantlets

The second group of 90 parental calli carried 232 green proliferating cell clusters that were transferred to shoot induction medium to stimulate the regeneration of plantlets. Of these, 100 cell clusters developed one or multiple shoots (**Supplementary Figure S5**). Because cell clusters were observed to be chimeric (**Supplementary Table S3**), we anticipated that different shoots from the same cell cluster could have different genotypes. Therefore, multiple shoots regenerated from individual cell clusters were harvested and transferred to root induction medium. Shoot regeneration continued over a period of seven months and Sanger sequencing data was obtained from leaves of 448 plantlets. Thirty-one (31%) cell clusters developed one or more shoots with an edited *CCR2* gene (**Supplementary Figure S5**). Of these, nineteen cell clusters yielded plantlets with different genotypes (i.e., different lines) (**Table 1**), indicating that, as anticipated, different sectors of chimeric cell clusters can develop into genetically different shoots. Detailed sequence analysis indicated that mutations at the *CCR2* gene could be classified into three categories or combinations thereof: i) small insertion or deletion (INDEL) mutations, ii) insertions originating from random *pCCR2-CRISPR* plasmid DNA fragments, and iii) insertions originating from genomic DNA fragments (**Table 2**, **Supplementary Figure S5**).

**Table 1:** Overview of shoots regenerated from green proliferating cell clusters with editing in the *PtaCCR2* gene.

Line	INDEL		Phenotype	Category	# Shoots	*NPTII*	*Cas9*
	*P. tremula* allele	*P. alba* allele					
**WT**	0	0	WT			–	–
**2.1**	+1;0	-1	Dwarfed	I	4	+	+
**2.5**	+75;0	+1;0	Dwarfed	I	1	+	+
**2.6**	+524	+1	Dwarfed	II	3		
**3**	+169	+321	Dwarfed	II	9		
**4.1**	+225	+1	Dwarfed	II	9		
**4.8**	0	0	WT	WT	1		
**5.1**	-1;0	-1;0	Dwarfed	I	2	–	–
**5.3**	0	0	WT	WT	1		
**7.1**	-1	0;-1	WT	I	1	–	–
**7.2**	0	0;+1	WT	I	1	–	–
**8.1**	+339	-2	Dwarfed	II + III	3		
**8.4**	0	0	WT	WT	2		
**12.1**	>+700	>+700	Dwarfed	II	2		
**12.3**	0	0	WT	WT	4		
**15.1**	+105;+74	0;-5;+1	Dwarfed	II	3		
**15.2**	-1	-1	Dwarfed	I	1	–	–
**18.1**	-1	0;-2	Dwarfed	I	5	+	+
**18.5**	-1	0;?	Dwarfed	I	1	+	+
**18.6**	-1	-1	Dwarfed	I	1	+	+
**18.7**	-1	0;+1	Dwarfed	I	1	+	+
**20.1**	+105	-5	Dwarfed	II	1		
**24.1**	+105	-5	Dwarfed	II	1		
**28.1**	+264	+73	Dwarfed	II	2		
**31.1**	0;+75	0	WT	II	1		
**31.2**	+163	0	WT	II	2		
**32.1**	0;60	0;+134	Dwarfed	II	2		
**32.3**	0	0	WT	WT	1		
**35**	+1	+121	Dwarfed	II	1		
**42.1**	+524;-1;-2;-4;-5	0;-3;0	Dwarfed	II	2		
**42.3**	+524	?	Dwarfed	II	3		
**42.5**	0	0	WT	WT	2		
**42.6**	+511;0	+1;+3;+4	Dwarfed	II	1		
**42.8**	+76;0	0	WT	III	3		
**43.1**	0	0	WT	WT	3		
**43.2**	0;+292	0;+163	WT	II	1		
**43.5**	0;+76	0	WT	III	1		
**44.2**	+76	+1	Dwarfed	III	2		
**45.1**	0	0	WT	WT	1		
**45.2**	+295	+163	Dwarfed	II	1		
**45.3**	+76	+4	Dwarfed	III	1		
**48.1**	+76	+1	Dwarfed	III	8		
**48.5**	+410	+1	Dwarfed	II	1		
**48.9**	?	+1	Dwarfed		1		
**84.1**	+142	+13	Dwarfed	II	7		
**84.7**	+76	0	WT	III	1		
**84.9**	+142	0	WT	II	1		
**116.1**	-1	-4	Dwarfed	I	1	+	
**116.2**	-3	-4	WT	I	1	+	+
**116.3**	+1	-4	Dwarfed	I	5	+	
**116.8**	chimera*	chimera*	Dwarfed		2		
**118.1**	-1	-1	Dwarfed	I	5	–	–
**120.1**	+163	+316	Dwarfed	II	2		
**125.1**	+73	+11	Dwarfed	II + III	10		
**125.4**	chimera*	+111	Dwarfed	II	3		
**136.1**	+1	?	Dwarfed		2		
**154.1**	+139;0	0;-3	WT	II + III	2		
**179.1**	+1	+111	Dwarfed	II	10		
**179.3**	0	0	WT	WT	1		
**179.5**	+1	0	WT	I	3	–	–
**179.9**	+1	0	WT	I	1	+	–
**179.15**	+1	+1	Dwarfed	I	1	+	–
**184**	?	-2	Dwarfed		1		
**189.1**	+357	+75	Dwarfed	II	2		
**191.1**	+224;0	0	WT	II	1		
**191.2**	0	0	WT	WT	1		
**191.3**	+1	+1	Dwarfed	I	7	+	+
**191.5**	0	+72	WT	II	1		

Line numbers are defined by the number of the harvested shoot, preceded by the number of the parental cell cluster (i.e. X.Y; shoot Y originating from cell cluster X). INDELs are represented on the *P. tremula* and *P. alba* allele, respectively, and are separated by a semicolon if multiple genotypes are observed (i.e. chimeric plants). *, chimeric INDELs were observed, but could not be resolved. ?, sequencing data could not be obtained for the respective allele. # shoots, the total number of regenerated shoots with an identical mutation in the *CCR2* gene. Genomic DNA of plantlets containing small INDELs at the *CCR2* target site (category I) were analyzed for the presence (+) or absence (-) of *NPTII* and *Cas9* DNA. Insertions of category II and III (insertions containing sequences of the *pCCR2-CRISPR* plasmid and genomic DNA, respectively) were not subjected to genotyping analyses for *NPTII* and *Cas9*. Note: as a single parental callus can develop multiple green proliferating cell clusters (**Figure 3**), it is possible that the manual separation of the cell clusters did not occur exclusively, as such that cells from one cell cluster might have ended up in a neighboring cell cluster, yielding shoots with identical genotypes (e.g. 20.1, and 24.1).

**Table 2:** Sequencing and genotyping summary of all regenerated shoots upon microprojectile-mediated delivery of the *pCCR2-CRISPR* plasmid in poplar calli.

	#Cell clusters	Shoot category	INDEL type of lines regenerated from cell clusters	#Shoots	*Cas9* and *NPTII*	
					-	+
					#Shoots	#Shoots
Cell clusters yielding one or more *CCR2*-edited shoots	31	I	INDELs	18	6	12
		II	Targeted insertion from *pCCR2*-*CRISPR* plasmid	26		
		III	Targeted insertion from genomic DNA	6		
		II + III	Cat. II + Cat. III	3		
		WT	WT	10		
						
Cell clusters yielding only *CCR2* WT shoots	69	WT	WT			
Total	100			63		

Because of the chimeric nature of the cell clusters, shoots with multiple genotypes, including WT, could be regenerated from a single cell cluster. Cell clusters that developed shoots could be classified into two classes: (i) cell clusters that gave rise to at least one shoot with mutations in the *CCR2* gene and (ii) cell clusters that gave solely rise to shoots with a WT *CCR2* gene. All the shoots derived from the cell clusters that had at least one shoot with mutations in the *CCR2* gene were further classified according to their different mutation types (category I, II, III, and WT). A diagram, illustrating a more elaborate origin and classification of the plant material, is depicted in **Supplementary Figure S5** together with relative percentages.

**Figure 5:**
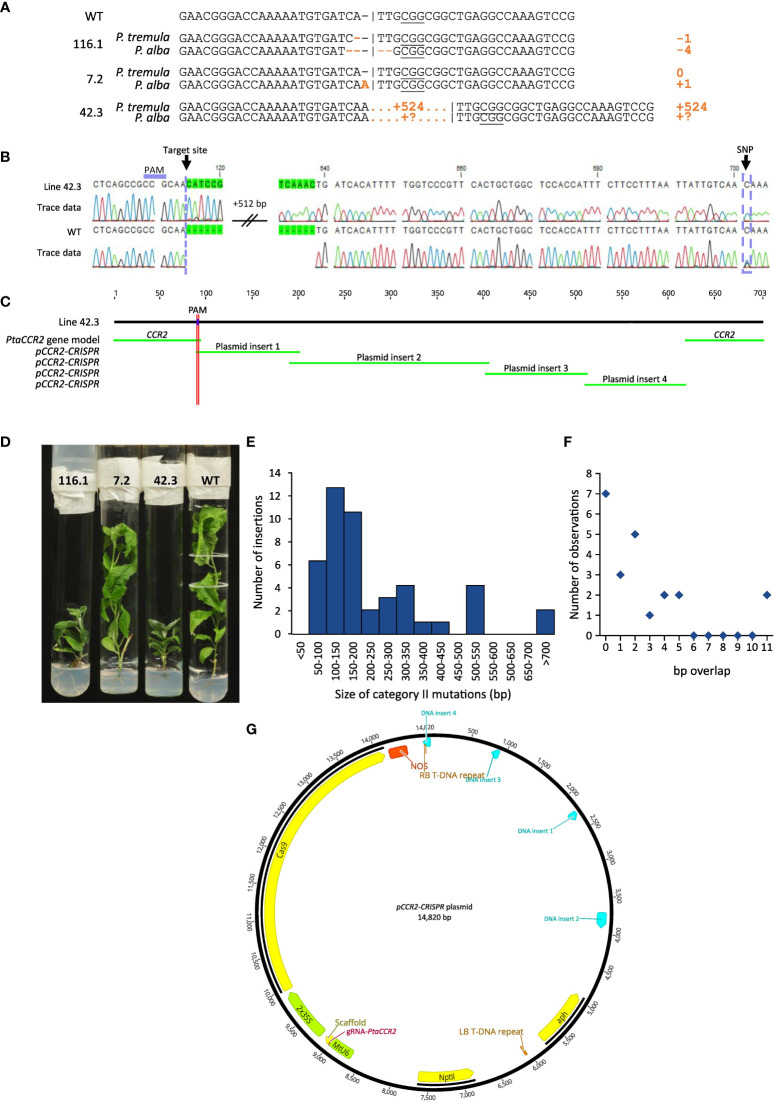
Sequence analysis of regenerated plantlets. Line numbers are defined by the number of the harvested shoot, preceded by the number of the parental cell cluster (i.e. X.Y; shoot Y originating from cell cluster X). **(A)** Cas9-induced gene editing in the *CCR2* gene. Different types of mutations are observed, ranging from simple INDELs as for lines 116.1 and 7.2 to large insertions as for line 42.3. **(B)** Sequence chromatogram illustrating a 524-bp insertion at the Cas9-induced DSB for line 42.3. By including information from single nucleotide polymorphisms (SNPs), insertions can be attributed to either the *P. tremula* or *P. alba* allele. In this example, a cytosine is observed at position 701, indicating that the 524-bp insertion is located on the *P. tremula* allele. The *P. alba* allele is represented by a guanine residue at this position. **(C)** BLAST analysis of a complete sequencing chromatogram against the *pCCR2-CRISPR* plasmid and the WT sequences yields multiple significant hits, suggesting the concatenation of multiple vector fragments and their subsequent integration into the Cas9 target site. **(D)** Phenotype of representative lines with simple INDELs (lines 116.1 and 7.2) and a large targeted insertion (line 42.3) compared to the WT. **(E)** Size distribution histogram of insertions in the Cas9 target site originating from the *pCCR2-CRISPR* plasmid (category II mutations). **(F)** Size distribution histogram of the overlap (in number of base pairs) between concatenated DNA fragments and/or the borders at the Cas9-induced DSB, as observed in lines 2.6, 42.3, 48.5, 120.1, and 189.1. **(G)** Concatenated DNA fragments integrated in the Cas9 target site of line 42.3 were mapped on the *pCCR2-CRISPR* plasmid to determine their origin. Hits are represented in light blue.

The first category contained 18 independent lines [or 34.0% of the mutated population (category I, II, III, and category II + III)] that contained mono-allelic or biallelic mutations in the *CCR2* gene (**Supplementary Figure S5**). As envisioned, biallelic knock-out mutations could be confirmed visually because the plantlets remained small and had dark-green leaves (**Figures 5A, D**). In contrast, mono-allelic (lines 7.1, 7.2, 179.5, and 179.9) or in-frame (line 116.2 and 154.1) mutations yielded plantlets that were indistinguishable from WT (**Figure 5D**), in line with the fact that the *CCR2* gene in *P. tremula* x *P. alba* is haplosufficient (De Meester et al., 2020) (**Supplementary Figure S6**). Notably, we identified one plantlet (line 116.2) without a noticeable phenotype but with a biallelic edit consisting of a 3- and 4-bp deletion in the *P. tremula* and *P. alba* allele, respectively. The in-frame deletion of 3 bp in the *P. tremula* allele resulted in the deletion of Ile-114 and did not affect the lignin amount of the plant (**Supplementary Figures S2, S6**). To further investigate whether regenerated plantlets of category I had transiently expressed the delivered DNA without incorporating a copy into the genome, we evaluated the presence of *Cas9*-coding DNA and the *NPTII* selectable marker gene using PCR. Molecular analyses indicated that 6 of the 18 lines (3.0% of all regenerated shoots) were free of *Cas9* and *NPTII* inserts (**Table 2**, **Supplementary Figure S5**).

The second category of mutations observed at the Cas9-induced DSB included DNA inserts originating from the *pCCR2-CRISPR* plasmid (26 lines or 49.1% of the mutated population) (**Supplementary Figure S5**). As observed for the sequenced cell clusters, DNA insertions originated from apparently random fragments of the plasmid (**Supplementary Figure S7**). In addition, concatenated DNA inserts, composed of multiple smaller fragments, typically less than 20 bp but up to 216 bp, were observed (**Figures 5C, G**, **Supplementary Figure S7B**). Detailed sequence analysis illustrated that each individual fragment had a few base pairs overlap with the next one or with the borders of the Cas9-induced DSB, suggesting that the integration and concatenation was achieved via microhomology-mediated end joining (MMEJ) (**Figure 5C, F**). The insertion sizes ranged from 58 bp up to more than 700 bp (**Figure 5E**). In accordance with reports in the literature, the majority of the characterized category II mutations (62%) were smaller than 200 bp (**Figure 5E**) (Svitashev et al., 2002). Yet, it must be noted that the distribution of insertions might be biased by the PCR effectiveness, as large inserts might not be amplified by the used PCR method. Notably, four lines (42.3, 48.9, 136.1, and 184) were identified in which only one allele could be sequenced, possibly because of technical limitations missing insertions or deletions of multiple kilobases and/or chromosomal rearrangements (**Table 1**, **Figure 5B**).

Category III mutations (6 lines or 11.3% of the mutated population) were characterized by the insertion of genomic DNA fragments at the Cas9-induced DSB (**Supplementary Figure S5**). In all six independent lines, an identical 76-bp insertion was observed that integrated into the Cas9 target site via MMEJ. BLAST analysis of the inserted DNA fragment against the poplar genome indicated that the insert originated from the multicopy endogenous 18S rRNA gene of poplar (D'Ovidio et al., 1990) (**Supplementary Table S5**).

In addition to the described mutation categories, we identified three lines (5.7% of the mutated population) that were characterized by the concurrent insertion of *pCCR2-CRISPR* plasmid sequences together with genomic DNA fragments (category II + III) (**Supplementary Figure S5**). One of these three lines (line 8.1) had a *P. tremula* allele that contained a concatenated sequence consisting of 125 bp originating from an intergenic region of *P. alba* chromosome 1, 51 bp originating from the *Cas9*-coding sequence of the *pCCR2-CRISPR* plasmid, and 167 bp originating from *E. coli* genomic DNA (**Supplementary Figure S8**). The latter sequence can likely be attributed to impurities originating from *pCCR2-CRISPR* plasmid preparation.

While characterizing the gene-edited population, we detected numerous lines displaying chimeric editing of the *CCR2* gene (**Table 1**). Such plants are characterized by the presence of genetically distinct cells within the same organism, while the phenotype of these plants is likely determined by the relative frequency of cells containing WT *CCR2* alleles. The occurrence of chimeric plants is frequently noted in the T0 generation of various plant species, including poplar (Ding et al., 2020). After multiple cycles of vegetative propagation, the frequency of *CCR2* chimeras decreased (**Supplementary Table S2**), in line with findings reported in the literature (Ding et al., 2020; Malabarba et al., 2020).

## 4 Discussion

The biolistic delivery of gene-editing reagents into plant cells appeared to occur at a low efficiency, presumably due to the limited number of cells that received microparticles upon bombardment. In this study, we visually screened 363 plants regenerated on non-selective medium and failed to identify plants with a characteristic *ccr2* knock-out phenotype. To overcome labor-intensive screenings, a temporary antibiotic selection was applied to enrich for transformed cells. After all, the transient expression of an antibiotic resistance gene together with *Cas9*-coding DNA could allow the precise editing of *CCR2* without necessarily incorporating the T-DNA into the genome of the plant. Using a 48-h selective treatment on kanamycin-containing medium, we obtained green proliferating cell clusters that gave rise to gene-edited shoots, some of which were potentially free of any plasmid DNA. The molecular screening of a subset of these cell clusters at an early stage in their development provided a good estimation of the gene editing.

Biallelic mutations in the *CCR2*-coding sequence were previously shown to induce a yield penalty and a dark-green coloration of the leaves, while monoallelic mutations in either the *P. tremula* or *P. alba* allele did not result in noticeable phenotypes (De Meester et al., 2020). In agreement with these previous findings, all lines that had biallelic mutations in the *CCR2* gene developed a dwarfed phenotype. We identified one line (line 116.2) containing a 3- and 4-bp deletion in the *P. tremula* and *P. alba* allele, respectively. The 4-bp deletion resulted in a frameshift, and thus knock-out mutation, in the *P. alba* allele. The 3-bp in-frame deletion in the *P. tremula* allele resulted in a structural change within the CCR2 protein that potentially altered its activity (**Supplementary Figure S2**). Using the same vector (and thus the same gRNA targeting the *CCR2* locus), De Meester and colleagues identified a line, called CCR2(-/*) line 12, which had a frameshift mutation (1-bp insertion) in the *P. tremula* allele, and a 3-bp deletion in the *P. alba* allele, resulting in the substitution of Ile-114 and Ala-115 for a Thr-114 (De Meester et al., 2020). This 3-bp deletion caused the allele to be haploinsufficient, which significantly enhanced the biomass processing properties of poplar due to a 10% reduction in lignin content (De Meester et al., 2020). However, the lignin amount in wood of line 116.2 was similar to that of the WT (**Supplementary Figure S6**). These results suggest that the new *P. alba CCR2* allele with the 3-bp deletion in line 116.2 was still haplosufficient.

In addition, we identified multiple lines that incorporated plasmid DNA and/or genomic DNA at the Cas9 target site. The use of physical force, inherent to the biolistic-mediated delivery of microparticles, often results in the shearing of vector and genomic DNA into a range of smaller fragments that can be incorporated in the process of DNA repair (Gorbunova and Levy, 1997). Several studies reported the integration of fragments of plasmid and/or genomic DNA into random locations in the genome upon biolistic-mediated transformation (Svitashev et al., 2002; Svitashev et al., 2015; Banakar et al., 2019; Liu et al., 2019). In this study, we observed DNA inserts originating from the *pCCR2-CRISPR* plasmid (category II mutations) and from endogenous genomic fragments (category III mutations) in 17.3% of all regenerated shoots (**Supplementary Figure S5**). To our knowledge, this is the highest frequency of targeted DNA inserts reported in plants in the literature. Likely, the frequency of targeted insertions is correlated with the amount of delivered DNA. These results also suggest that the integration of donor DNA can be steered on purpose and might thus provide a neat alternative for homology-directed repair (HDR) strategies that are currently extremely low in efficiency in plants, as was shown in maize (Svitashev et al., 2015). Although promising, genomic rearrangements and gene disruptions are frequently observed upon biolistic-mediated transformation (Svitashev et al., 2002; Liu et al., 2019). Therefore, it is advised to perform whole-genome sequencing to eliminate plantlets with undesired genomic rearrangements while selecting promising lines that contain the desired insert before proceeding toward applications.

## 5 Conclusions

In this study, we investigated the delivery of CRISPR DNA via microparticles to assess its potential for developing transgene-free, gene-edited trees and integrating donor DNA at specific target sites. To this end, we used a temporary selection method and succeeded in generating poplars that contain small INDEL mutations at the Cas9 target site. PCR analyses indicated that up to 3.0% of these lines must have transiently expressed the CRISPR cassette without integrating it into the genome of the plant, a frequency that is at least 10-fold higher as compared to a previously reported transient transformation strategy in apple tree (Charrier et al., 2019), and as efficient as earlier reports in tomato (Jacobs et al., 2017). The ability to develop gene-edited trees that are free of *Cas9*-coding DNA is essential to translate basic research from the lab to the field.

In addition, our method is characterized by the frequent insertion of plasmid DNA at the Cas9-induced DSB, a phenomenon which can be used for precise DNA insertions and might overcome the low-frequency limitation from which HDR is currently suffering. Via this method, we provide the science community with additional tools to speed up the development of highly productive trees that are suited as a resource in the bio-economy (Vanholme et al., 2013; De Meester et al., 2022).

## Data availability statement

The original contributions presented in the study are included in the article/**Supplementary Material**. Further inquiries can be directed to the corresponding author.

## Author contributions

LH: Conceptualization, Formal Analysis, Funding acquisition, Investigation, Methodology, Writing – original draft, Writing – review & editing, Data curation. JD: Conceptualization, Investigation, Writing – review & editing. RV: Conceptualization, Funding acquisition, Investigation, Supervision, Writing – review & editing. WB: Conceptualization, Funding acquisition, Investigation, Supervision, Writing – review & editing.
