# Supplementary material for: Microparticle-mediated CRISPR DNA delivery for genome editing in poplar
Source: Front Plant Sci. 2023 Nov 13;14:1286663. doi: 10.3389/fpls.2023.1286663 (PMC10679337; doi:10.3389/fpls.2023.1286663)
Supplement: Supplementary file 1 [file DataSheet_1.pdf]

Microparticle-mediated CRISPR DNA delivery for transgene-free genome editing in poplar

Lennart Hoengenaert[1,2], Jan Van Doorsselaere[3], Ruben Vanholme[1,2,†], and Wout Boerjan[1,2,*,†]

Supplemental data includes

- **Fig. S1-S8**
- **Table S1-S5**
- **Supplementary references**

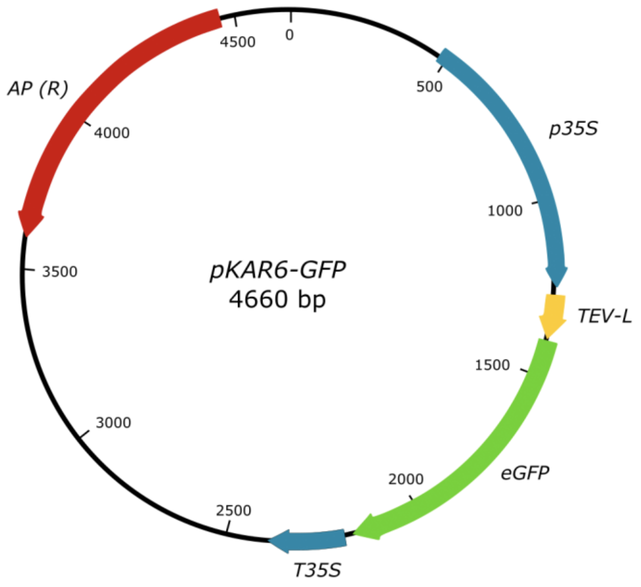

Fig. S1: Schematic representation of the *pKAR6-GFP* plasmid used for transformation validation. A 4.6-kb reporter plasmid was used to validate the transformation of *P. tremula x P. alba* callus. AP (R), Ampicillin resistance marker; *p35S:* CaMV-35S promoter; *TEV-L*, translation enhancer from tobacco etch virus (TEV); *eGFP*, enhanced *GFP* followed by the KDEL ER retention signal; *T35S:* CaMV-35S terminator.

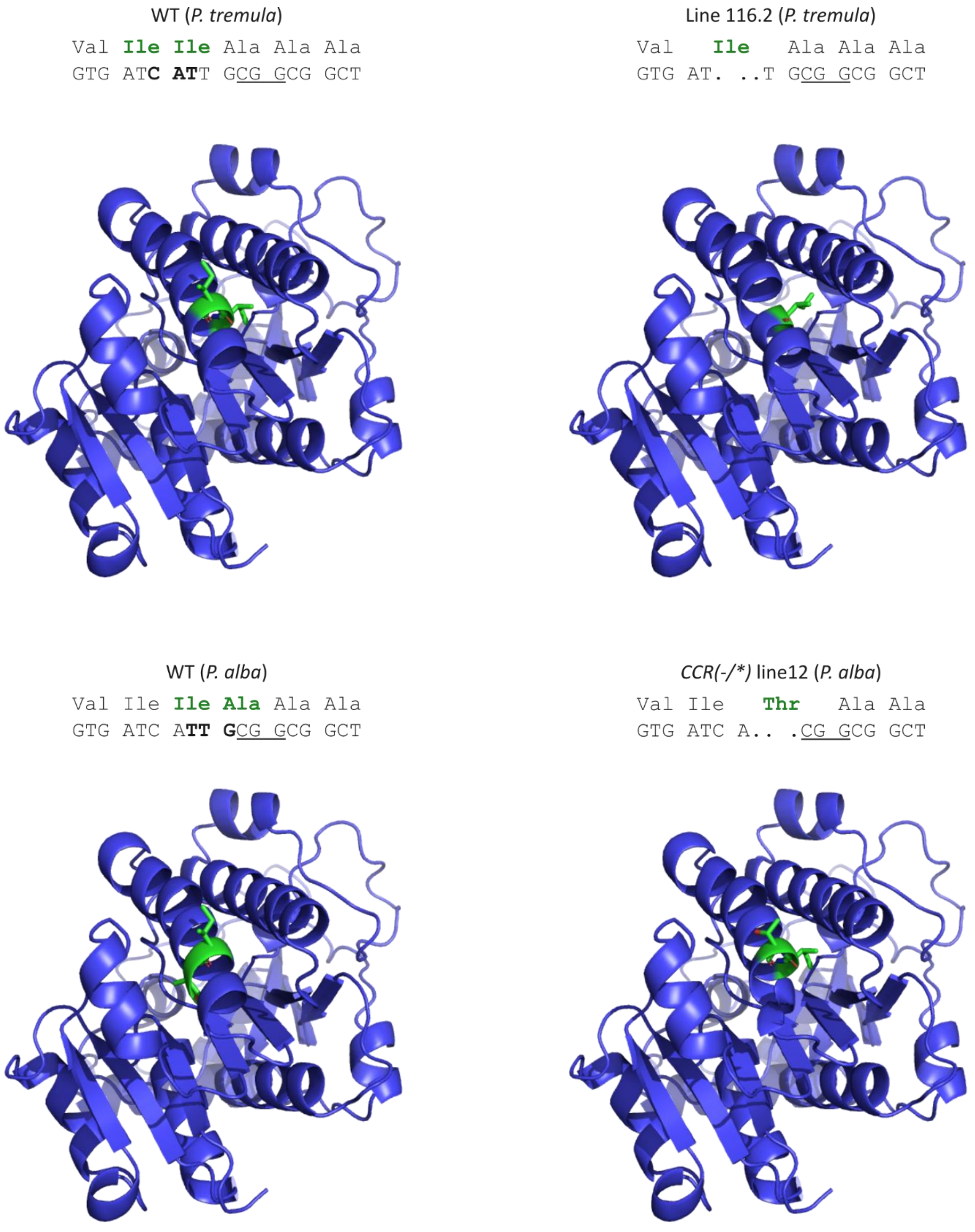

Fig. S2: Allele-specific models of the CCR2 protein of WT, line 116.2 (-3/-4), and *CCR2(-/*)* line 12 (+1/-3). Using the *P. tremula* and *P. alba* nucleotide sequences, protein models were created for WT CCR2 protein. Mutated CCR2 proteins of lines 116.2 and *CCR2(-/*)* line 12 (De Meester et al., 2020) were modeled using the respective mutated nucleotides sequences. The 3-bp deletion in line 116.2 resulted in the deletion of Ile-114, while the 3-bp deletion in *CCR2(-/*)* line 12 resulted in the substitution of Ile-114 and Ala-115 into Thr-114. Affected nucleotides are marked in bold, while their respective amino acids are marked in green. The PAM sequence is underlined.

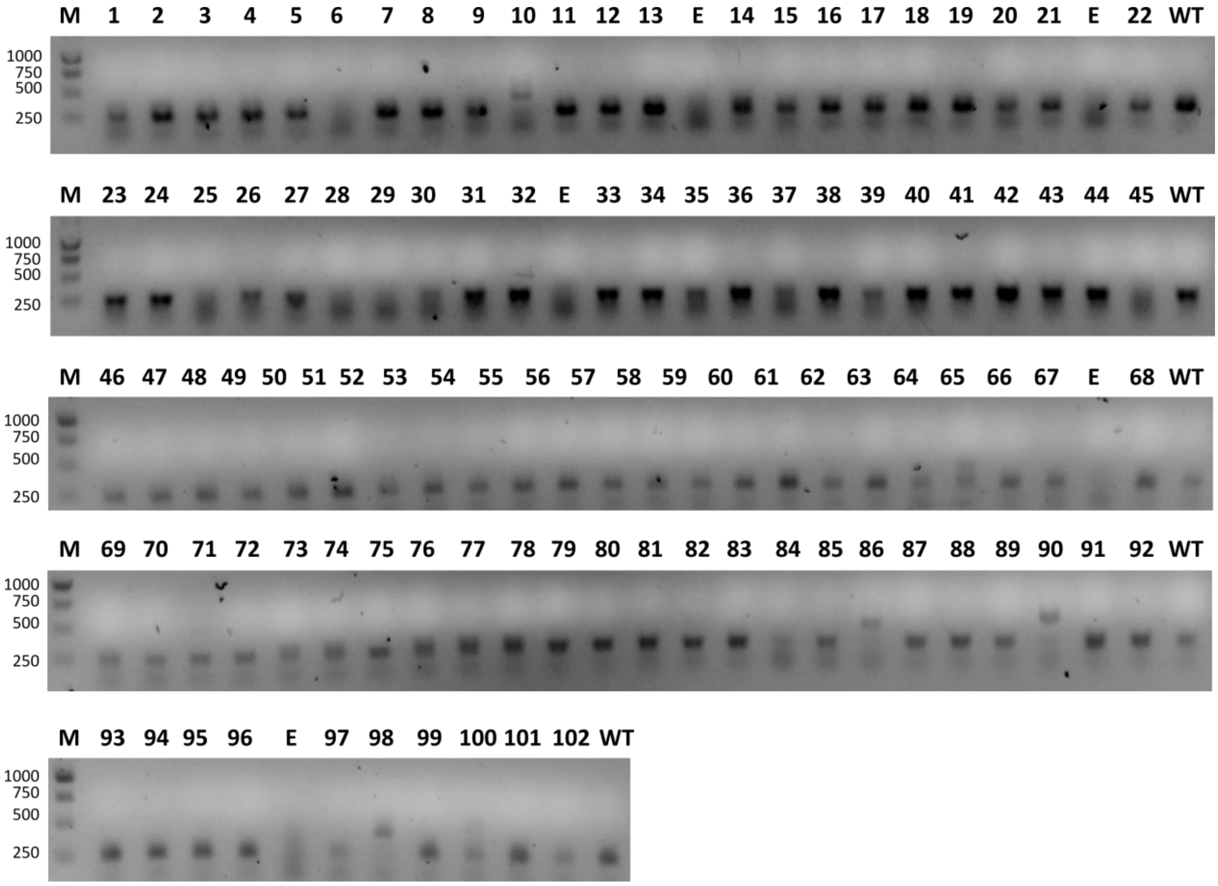

Fig. S3: Amplification of the *PtaCCR2* target region in green proliferating cell clusters. Genomic DNA extracts were used to amplify the *PtaCCR2* target region using gene-specific primers, followed by migration through a 1.2% agarose gel. The theoretical size of the *PtaCCR2* amplicon is 249 bp. For samples indicated with the letter E, no PCR amplicon could be obtained.

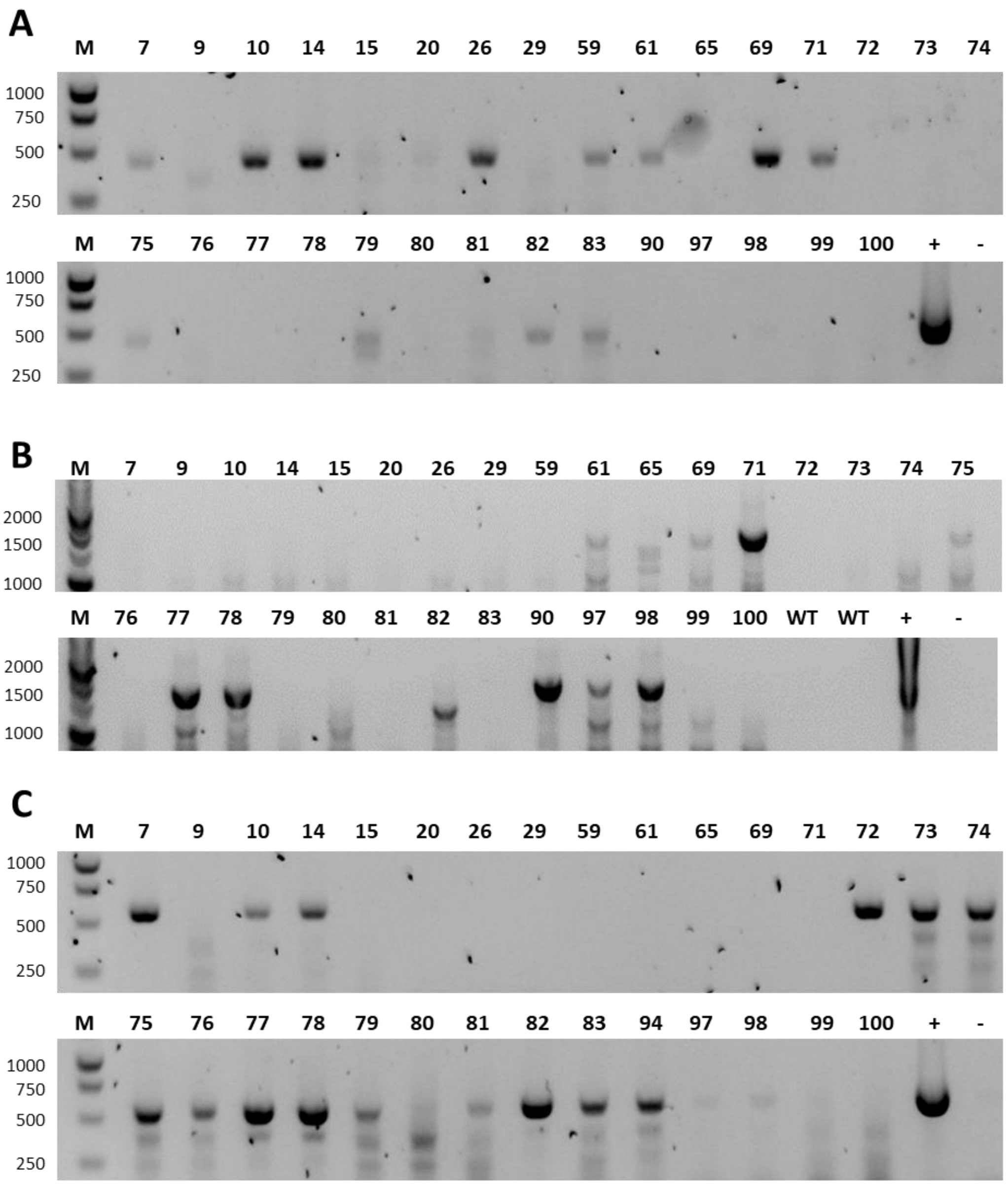

Fig. S4: Genotyping of cell clusters with significant gene editing. Cell clusters with significant gene editing were subjected to PCR-based genotyping analyses. **A)** *Cas9* amplicon, theoretical size: 436 bp. **B)** *CCR2-gRNA* amplicon, theoretical size: 1852 bp. **C)** *NPTII* amplicon, theoretical size: 558 bp.

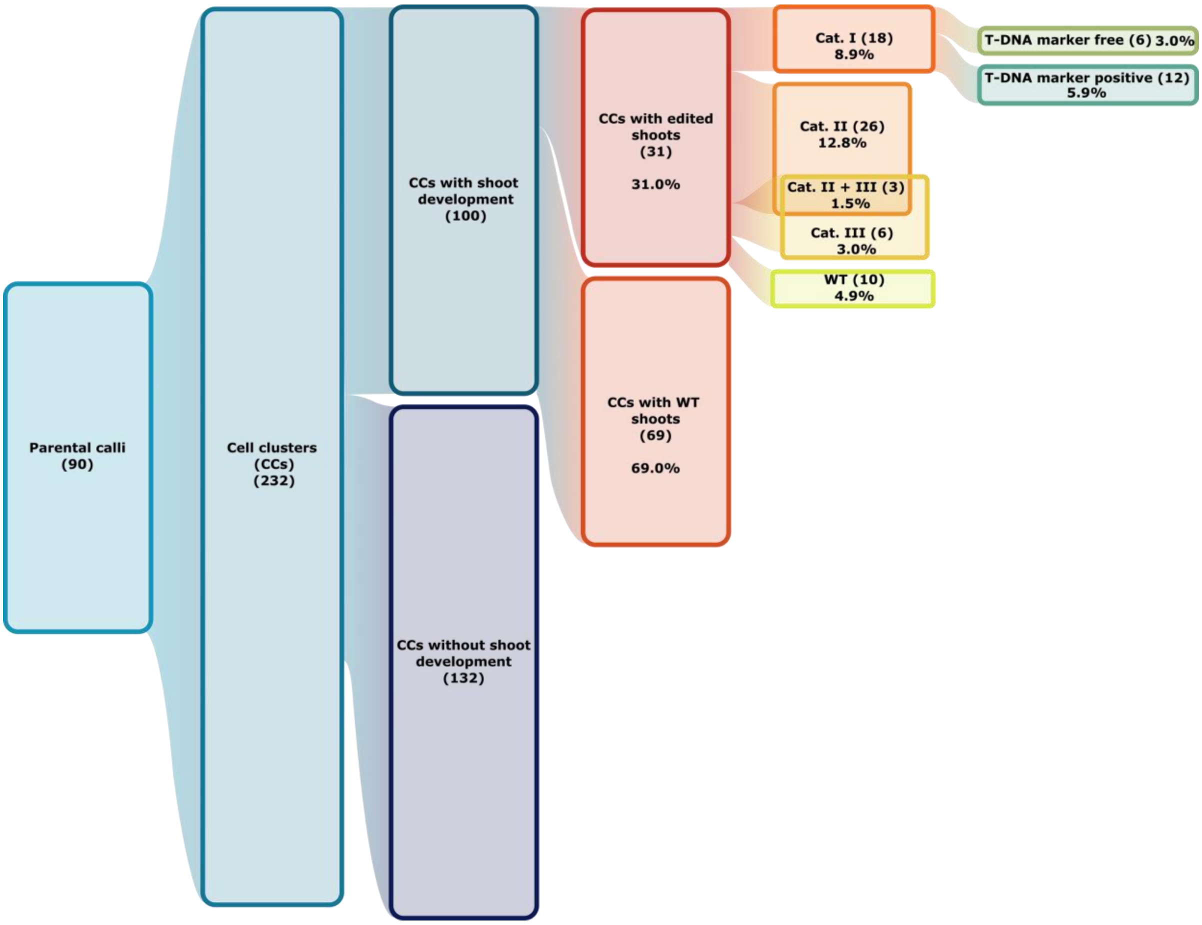

Fig. S5: Diagram summarizing the cultivation of cell clusters and the regenerated shoots after microprojectile-mediated delivery of the *pCCR2-CRISPR* plasmid in poplar calli. Upon transformation followed by a short selective treatment, green proliferating cell clusters (CCs) originated on parental calli. These CCs were cultivated on shoot-inducing medium over a period of 7 months. Because of the chimeric nature of the CCs, shoots of multiple genotypes, including WT, could be regenerated from a single CC. As such, CCs that developed shoots could be classified in two classes: CCs that produced at least one shoot with editing in the *CCR2* gene, and CCs that solely produced WT shoots. CCs yielding at least one edited shoot were further classified according to their mutation type (Cat. I, simple INDELS; Cat. II, insertions originating from the *pCCR2-CRISPR* plasmid; Cat. III, insertions originating from genomic DNA; and WT) and the absence or presence of T-DNA markers. Percentages represents the portion of shoots within a specific category in relation to the overall number of harvested shoots (including both WT and *CCR2*-edited shoots). Right three columns are not to scale.

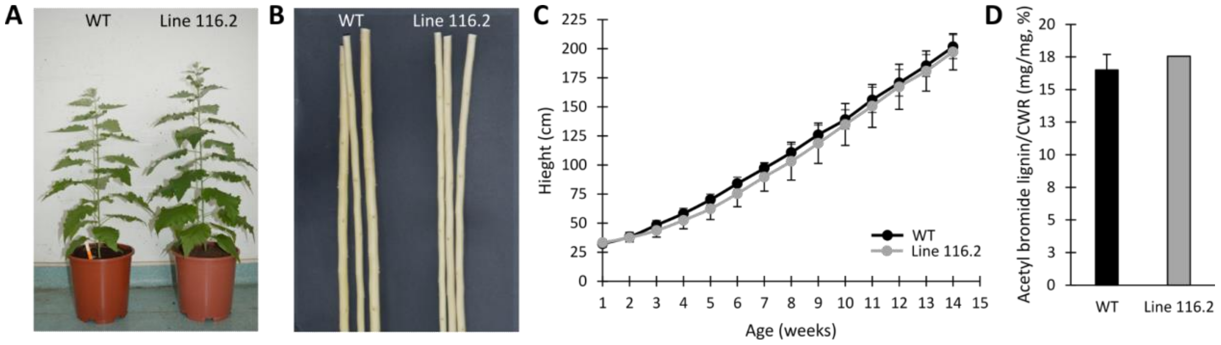

Fig. S6: Characterization of line 116.2 containing a 3- and 4-bp deletion in the *P. tremula* and *P. alba* allele, respectively. A) A representative clonal replicate of line 116.2 grown under greenhouse conditions next to the wild type (WT) at 1 m height. **B)** Upon debarking of the main stem, no red xylem coloration was observed for line 116.2. Red xylem is a phenomenon that is typically observed in *CCR*-downregulated poplar (Leplé et al., 2007; Ralph et al., 2008; De Meester et al., 2020). **C)** Growth analysis of line 116.2 compared to WT under greenhouse conditions. No significant difference was detected at any of the measured time points (n = 6 for each genotype). **D)** To investigate whether the in-frame deletion of 3 bp affected the CCR2 protein activity, the lignin amount was evaluated. The acetyl bromide lignin amount of line 116.2 was determined to be 17.56% of cell wall residue (CWR), which lies within one standard deviation of the 16.51% average of the WT (n_{WT} = 7, $n_{line\ 116.2}$ = 1), suggesting that the altered CCR2 protein did not change the lignin amount in the plant. The single and mutated *P. tremula CCR2* allele was still sufficient to support a normal *CCR2* gene function.

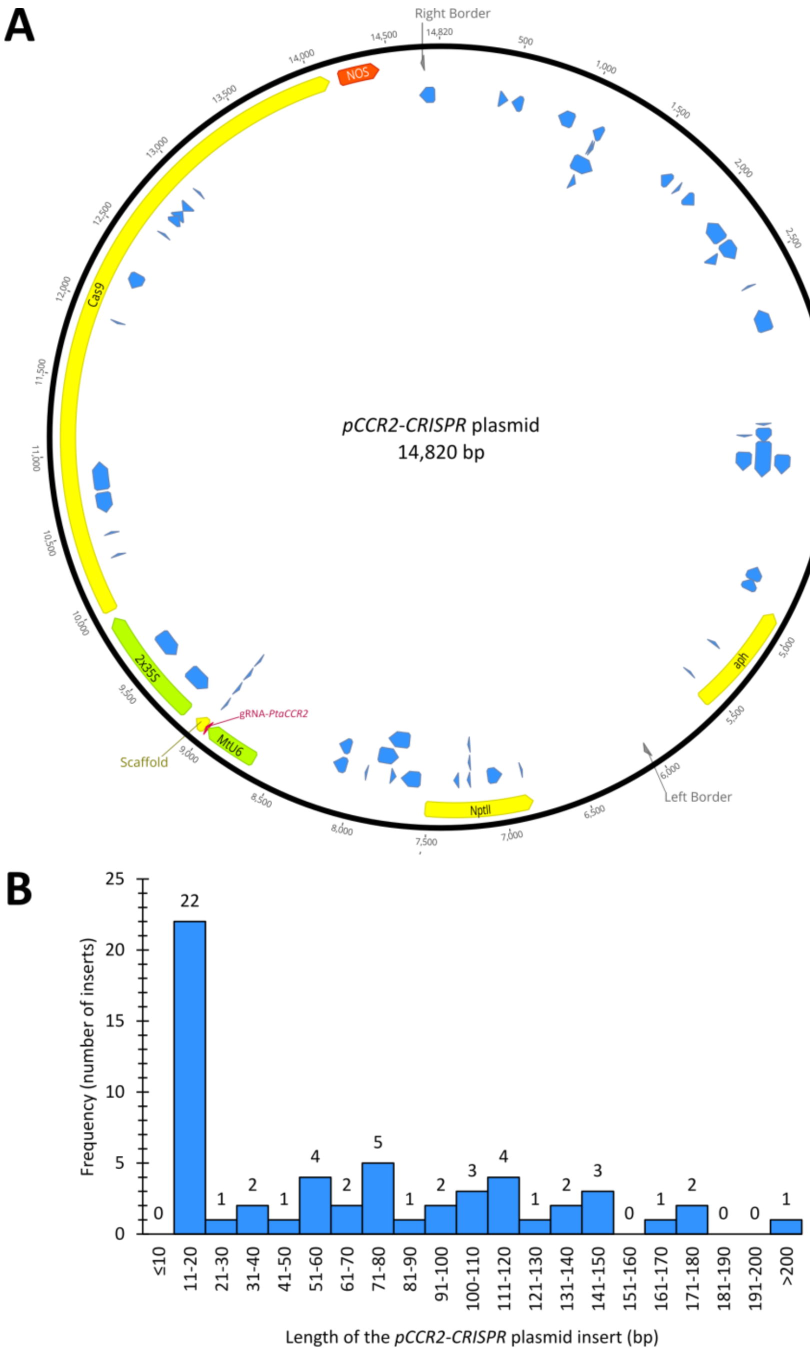

Fig. S7: Origin of *pCCR2-CRISPR* plasmid insertions at the Cas9 target site. A) The original location of plasmid DNA fragments inserted at the Cas9 target site (category II mutations, Table 1) mapped along the *pCCR2-CRISPR* plasmid. Blue arrows indicate the original location of the DNA fragments and their orientation. Some lines were left out of the analysis as their sequence chromatogram was too complex. **B)** Distribution of the length of the *pCCR2-CRISPR* plasmid inserts depicted in panel A.

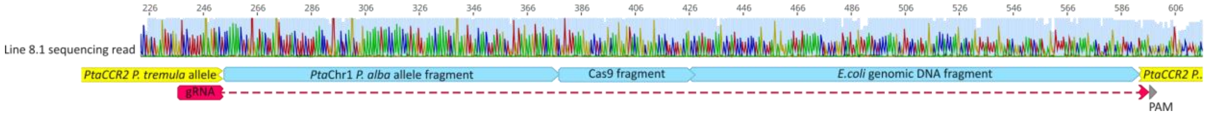

Fig. S8: Characterization of the DNA insertion in the *PtaCCR2* gene in line 8.1. The *P. tremula* allele of the *PtaCCR2* gene was amplified using gene-specific primers and subsequently analyzed through Sanger sequencing. At the Cas9 target site, a concatenated 339 bp DNA sequence was observed, consisting of an intergenic region of the chromosome 1 (Chr1) *P. alba* allele (125 bp), a fragment of the Cas9 coding sequence of the *pCCR2-CRISPR* plasmid (51 bp), and an *E. coli* genomic DNA fragment (167 bp). The latter sequence can likely be attributed to impurities originating from *pCCR2-CRISPR* plasmid preparation. Individual DNA fragments of the targeted insertion overlap a few base pairs with each other and the borders of the Cas9-induced DSB, suggesting that the integration and concatenation of the individual fragments was achieved via MMEJ. The genomic sequence of the *PtaCCR2 P. tremula* allele is highlighted in yellow, while individual DNA insertions at the Cas9 target site are highlighted in blue. The gRNA sequence and PAM recognition site are highlighted in red and grey, respectively.

Table S1: Media composition used for the cultivation of *P. tremula x P. alba* plantlets. MS, Murashige and Skoog basal salt mixture; IAA, indole-3-acetic acid; NAA, 1-naphthalene acetic acid; 2iP, 2-isopentenyladenine; TDZ, thidiazuron. The composition of these media are described in Leplé et al. (1992).

	M1/2WT	M1	M3	M3K
Purified water	1 L	1 L	1 L	1 L
MS	2.2 g	4.4 g	4.4 g	4.4 g
L-cysteine (10 mg/mL)	100 µL	100 µL	100 µL	100 µL
Glutamine	0.2 g	0.2 g	0.2 g	0.2 g
Sucrose	20 g	30 g	30 g	30 g
Plant agar (Duchefa)	5.5 g	6 g	6 g	6 g
Vitamins (Morel & Wetmore)	10 mL	10 mL	10 mL	10 mL
IAA (0.5 mg/mL)	1 mL			
NAA (0.5 mg/mL)		4 mL		
2iP (0.5 mg/mL)		2 mL		
TDZ (0.5 mg/mL)			40 µL	40 µL
Kanamycin (50 mg/mL)				2 mL

Table S2: Characterization of regenerated shoots from the *PtaCCR2* gene-edited population after multiple cycles of vegetative propagation. A selection of lines from the gene-edited population (Table 1) was vegetatively propagated to investigate the potential dechimerization of the *CCR2* locus upon successive cycles of shoot regeneration. INDELs are represented on the *P. tremula* and *P. alba* allele, respectively, and are separated by a semicolon if multiple genotypes are observed (i.e. chimeric plants). ?, sequencing data could not be obtained for the respective allele. Lines are classified according to their mutation at the Cas9 target site: I, INDELs; II, insertions originating from the *pCCR2-CRISPR* plasmid sequence; III, insertions originating from genomic DNA fragments. Genomic DNA extracts were analyzed for the presence (+) or absence (-) of the *NPTII* and *Cas9* DNA.

**Line**	**INDEL**		**Category**	***NPTII***	***Cas9***
	P.tremula allele	*P.alba* allele			
**WT**	0	0	WT	-	-
**2.1**	?	+1		+	+
**4.1**	+225	+1	II	-	-
**4.8**	0	0	WT	+	+
**7.1**	0	0	WT	-	-
**7.2**	0	0	WT	-	-
**8.1**	+338	-2	II + III	-	-
**8.4**	0	0	WT	-	-
**15.1**	+74	-5	II	-	-
**18.6**	-1	?		+	+
**28.1**	+264	+73	II	-	-
**31.1**	0	0	WT	-	-
**32.1**	+60	+134	II	-	-
**42.8**	0	0	WT	-	-
**43.1**	0	0	WT	-	+
**43.2**	0	0	WT	+	-
**43.5**	0	0	WT	-	-
**45.3**	+76	?	III	+	+
**48.1**	+76	+1	III	+	+
**84.9**	+142	0	II	+	+
**116.2**	-3	-4	I	+	+
**116.3**	+1	-4	I	-	-
**118.1**	-1	-1	I	-	-
**125.1**	+73	+111	II + III	-	-
**154.1**	+139	-3	II + III	+	+
**191.1**	0	0	WT	+	+
**191.2**	0	0	WT	-	-
**191.3**	+1	+1	I	+	+

Table S3: Contribution of INDELs in proliferating cell clusters with significant gene editing in the *CCR2* locus. Synthego's ICE analysis tool was used to determine the sequence profile. The quality of the predicted INDELs, together with their respective contribution is bundled in the R-squared value. Because the current algorithm of Synthego's ICE is not optimized for the detection of DNA insertions larger than 40 bp, such larger inserts were deduced manually and indicated with an asterisk (*). No sequence information could be obtained for cell cluster 86 due to the poor quality of the chromatogram. Nonetheless, the PCR amplicon showed a higher molecular weight on gel (Fig. S3), suggesting the insertion of a stretch of nucleotides.

<table>
<tr><th></th><th>INDEL</th><th>Contribution (%)</th><th>R Squared</th><th>Sequence</th></tr>
<tr><td>WT</td><td>0</td><td>100%</td><td>1</td><td>CGGACTTTGGCCTCAGCCGCCGCAA|TGATCACATTTTTGGTCCCGTTCACTGCTGGCT</td></tr>
<tr><td>Cell cluster 6</td><td>-3</td><td>85%</td><td>0</td><td>CGGACTTTGGCCTCAGCCGCCGC--|-GATCACATTTTTGGTCCCGTTCACTGCTGGCT</td></tr>
<tr><td rowspan="2">Cell cluster 7</td><td>0</td><td>99%</td><td rowspan="2">1</td><td>CGGACTTTGGCCTCAGCCGCCGCAA|TGATCACATTTTTGGTCCCGTTCACTGCTGGCT</td></tr>
<tr><td>-1</td><td>1%</td><td>CGGACTTTGGCCTCAGCCGCCGCAA|-GATCACATTTTTGGTCCCGTTCACTGCTGGCT</td></tr>
<tr><td rowspan="2">Cell cluster 9</td><td>-1</td><td>92%</td><td rowspan="2">0.99</td><td>CGGACTTTGGCCTCAGCCGCCGCA-|TGATCACATTTTTGGTCCCGTTCACTGCTGGCT</td></tr>
<tr><td>0</td><td>7%</td><td>CGGACTTTGGCCTCAGCCGCCGCAA|TGATCACATTTTTGGTCCCGTTCACTGCTGGCT</td></tr>
<tr><td>Cell cluster 10*</td><td>+142</td><td></td><td></td><td>CGGACTTTGGCCTCAGCCGCCGCA-|n_{142}ATGATACATTTTTGGTCCCGTTCACTGCTGGCT</td></tr>
<tr><td rowspan="3">Cell cluster 14</td><td>-11</td><td>74%</td><td rowspan="3">0.98</td><td>CGGACTTTGGCCTCAG---------|--ATCACATTTTTGGTCCCGTTCACTGCTGGCT</td></tr>
<tr><td>+1</td><td>21%</td><td>CGGACTTTGGCCTCAGCCGCCGCAA|nTGATCACATTTTTGGTCCCGTTCACTGCTGGCT</td></tr>
<tr><td>-11</td><td>3%</td><td>CGGACTTTGGCCTCAGCCG------|-----ACATTTTTGGTCCCGTTCACTGCTGGCT</td></tr>
<tr><td rowspan="2">Cell cluster 15</td><td>+1</td><td>78%</td><td rowspan="2">0.99</td><td>CGGACTTTGGCCTCAGCCGCCGCAA|nTGATCACATTTTTGGTCCCGTTCACTGCTGGCT</td></tr>
<tr><td>0</td><td>21%</td><td>CGGACTTTGGCCTCAGCCGCCGCAA|TGATCACATTTTTGGTCCCGTTCACTGCTGGCT</td></tr>
<tr><td rowspan="2">Cell cluster 20</td><td>0</td><td>95%</td><td rowspan="2">0.99</td><td>CGGACTTTGGCCTCAGCCGCCGCAA|TGATCACATTTTTGGTCCCGTTCACTGCTGGCT</td></tr>
<tr><td>-1</td><td>4%</td><td>CGGACTTTGGCCTCAGCCGCCGCAA|-GATCACATTTTTGGTCCCGTTCACTGCTGGCT</td></tr>
<tr><td rowspan="4">Cell cluster 26</td><td>-1</td><td>53%</td><td rowspan="4">0.98</td><td>CGGACTTTGGCCTCAGCCGCCGCA-|TGATCACATTTTTGGTCCCGTTCACTGCTGGCT</td></tr>
<tr><td>-2</td><td>35%</td><td>CGGACTTTGGCCTCAGCCGCCGCA-|-GATCACATTTTTGGTCCCGTTCACTGCTGGCT</td></tr>
<tr><td>-2</td><td>7%</td><td>CGGACTTTGGCCTCAGCCGCCGCAA|--ATCACATTTTTGGTCCCGTTCACTGCTGGCT</td></tr>
<tr><td>0</td><td>3%</td><td>CGGACTTTGGCCTCAGCCGCCGCAA|TGATCACATTTTTGGTCCCGTTCACTGCTGGCT</td></tr>
<tr><td rowspan="2">Cell cluster 29</td><td>0</td><td>94%</td><td rowspan="2">0.98</td><td>CGGACTTTGGCCTCAGCCGCCGCAA|TGATCACATTTTTGGTCCCGTTCACTGCTGGCT</td></tr>
<tr><td>-1</td><td>4%</td><td>CGGACTTTGGCCTCAGCCGCCGCAA|-GATCACATTTTTGGTCCCGTTCACTGCTGGCT</td></tr>
</table>

Table S3 (cont.): Contribution of INDELs in proliferating cell clusters with significant gene editing in the *CCR2* locus.

	INDEL	Contribution (%)	R Squared	Sequence
Cell cluster 30	0	60%	0.98	CGGACTTTGGCCTCAGCCGCCGCAA|TGATCACATTTTTGGTCCCGTTCACTGCTGGCT
	-1	38%		CGGACTTTGGCCTCAGCCGCCGCAA|-GATCACATTTTTGGTCCCGTTCACTGCTGGCT
Cell cluster 59	+1	81%	0.98	CGGACTTTGGCCTCAGCCGCCGCAA|nTGATCACATTTTTGGTCCCGTTCACTGCTGGCT
	0	17%		CGGACTTTGGCCTCAGCCGCCGCAA|TGATCACATTTTTGGTCCCGTTCACTGCTGGCT
Cell cluster 61	-2	40%	0.96	CGGACTTTGGCCTCAGCCGCCGCAA|--ATCACATTTTTGGTCCCGTTCACTGCTGGCT
	-1	35%		CGGACTTTGGCCTCAGCCGCCGCA-|TGATCACATTTTTGGTCCCGTTCACTGCTGGCT
	0	21%		CGGACTTTGGCCTCAGCCGCCGCAA|TGATCACATTTTTGGTCCCGTTCACTGCTGGCT
Cell cluster 65*	-1	40%	0.71	CGGACTTTGGCCTCAGCCGCCGCAA|-GATCACATTTTTGGTCCCGTTCACTGCTGGCT
	+46			CGGACTTTGGCCTCAGCCGCCGCA-|n$_{46}$ATGATACATTTTTGGTCCCGTTCACTGCTGGCT
Cell cluster 68	-3	48%	0.97	CGGACTTTGGCCTCAGCCGCCGCAA|---TCACATTTTTGGTCCCGTTCACTGCTGGCT
	-2	47%		CGGACTTTGGCCTCAGCCGCCGCAA|--ATCACATTTTTGGTCCCGTTCACTGCTGGCT
	-3	2%		CGGACTTTGGCCTCAGCCGCCGC--|-GATCACATTTTTGGTCCCGTTCACTGCTGGCT
Cell cluster 69	-4	92%	0.99	CGGACTTTGGCCTCAGCCGCCGCA-|---TCACATTTTTGGTCCCGTTCACTGCTGGCT
	0	7%		CGGACTTTGGCCTCAGCCGCCGCAA|TGATCACATTTTTGGTCCCGTTCACTGCTGGCT
Cell cluster 71	+1	81%	0.95	CGGACTTTGGCCTCAGCCGCCGCAA|nTGATCACATTTTTGGTCCCGTTCACTGCTGGCT
	0	12%		CGGACTTTGGCCTCAGCCGCCGCAA|TGATCACATTTTTGGTCCCGTTCACTGCTGGCT
	-10	1%		CGGACTTTGGCCTCAGCCGCCGCAA|----------TTTGGTCCCGTTCACTGCTGGCT
	-21	1%		CGGACTTTGGCCTCAGCCG------|---------------TCCCGTTCACTGCTGGCT
Cell cluster 72	0	84%	0.99	CGGACTTTGGCCTCAGCCGCCGCAA|TGATCACATTTTTGGTCCCGTTCACTGCTGGCT
	+1	6%		CGGACTTTGGCCTCAGCCGCCGCAA|nTGATCACATTTTTGGTCCCGTTCACTGCTGGCT
	-2	5%		CGGACTTTGGCCTCAGCCGCCGCAA|--ATCACATTTTTGGTCCCGTTCACTGCTGGCT
	-1	4%		CGGACTTTGGCCTCAGCCGCCGCAA|-GATCACATTTTTGGTCCCGTTCACTGCTGGCT
Cell cluster 73	-2	64%	0.93	CGGACTTTGGCCTCAGCCGCCGCAA|--ATCACATTTTTGGTCCCGTTCACTGCTGGCT
	-8	27%		CGGACTTTGGCCTCAGCCGCCG---|-----ACATTTTTGGTCCCGTTCACTGCTGGCT
	0	2%		CGGACTTTGGCCTCAGCCGCCGCAA|TGATCACATTTTTGGTCCCGTTCACTGCTGGCT

Table S3 (cont.): Contribution of INDELs in proliferating cell clusters with significant gene editing in the *CCR2* locus.

	INDEL	Contribution (%)	R Squared	Sequence
Cell cluster 74	-2	68%	0.94	CGGACTTTGGCCTCAGCCGCCGCAA|--ATCACATTTTTGGTCCCGTTCACTGCTGGCT
	-8	24%		CGGACTTTGGCCTCAGCCGCCG---|-----ACATTTTTGGTCCCGTTCACTGCTGGCT
	0	2%		CGGACTTTGGCCTCAGCCGCCGCAA|TGATCACATTTTTGGTCCCGTTCACTGCTGGCT
Cell cluster 75	+1	99%	0.99	CGGACTTTGGCCTCAGCCGCCGCAA|nTGATCACATTTTTGGTCCCGTTCACTGCTGGCT
Cell cluster 76	-2	61%	0.92	CGGACTTTGGCCTCAGCCGCCGCAA|--ATCACATTTTTGGTCCCGTTCACTGCTGGCT
	-8	28%		CGGACTTTGGCCTCAGCCGCCG---|-----ACATTTTTGGTCCCGTTCACTGCTGGCT
	-2	2%		CGGACTTTGGCCTCAGCCGCCGC--|TGATCACATTTTTGGTCCCGTTCACTGCTGGCT
	0	1%		CGGACTTTGGCCTCAGCCGCCGCAA|TGATCACATTTTTGGTCCCGTTCACTGCTGGCT
Cell cluster 77	-2	50%	0.89	CGGACTTTGGCCTCAGCCGCCGCAA|--ATCACATTTTTGGTCCCGTTCACTGCTGGCT
	-8	37%		CGGACTTTGGCCTCAGCCGCCG---|-----ACATTTTTGGTCCCGTTCACTGCTGGCT
	0	1%		CGGACTTTGGCCTCAGCCGCCGCAA|TGATCACATTTTTGGTCCCGTTCACTGCTGGCT
	-5	1%		CGGACTTTGGCCTCAGCCGCCG---|--ATCACATTTTTGGTCCCGTTCACTGCTGGCT
Cell cluster 78	-2	50%	0.90	CGGACTTTGGCCTCAGCCGCCGCAA|--ATCACATTTTTGGTCCCGTTCACTGCTGGCT
	-8	36%		CGGACTTTGGCCTCAGCCGCCG---|-----ACATTTTTGGTCCCGTTCACTGCTGGCT
	0	4%		CGGACTTTGGCCTCAGCCGCCGCAA|TGATCACATTTTTGGTCCCGTTCACTGCTGGCT
Cell cluster 79	0	66%	0.98	CGGACTTTGGCCTCAGCCGCCGCAA|TGATCACATTTTTGGTCCCGTTCACTGCTGGCT
	-1	17%		CGGACTTTGGCCTCAGCCGCCGCAA|-GATCACATTTTTGGTCCCGTTCACTGCTGGCT
	+1	7%		CGGACTTTGGCCTCAGCCGCCGCAA|nTGATCACATTTTTGGTCCCGTTCACTGCTGGCT
	+2	5%		CGGACTTTGGCCTCAGCCGCCGCAA|nnTGATCACATTTTTGGTCCCGTTCACTGCTGGCT
	-1	3%		CGGACTTTGGCCTCAGCCGCCGCA-|TGATCACATTTTTGGTCCCGTTCACTGCTGGCT
Cell cluster 80	+1	34%	0.96	CGGACTTTGGCCTCAGCCGCCGCAA|nTGATCACATTTTTGGTCCCGTTCACTGCTGGCT
	-1	33%		CGGACTTTGGCCTCAGCCGCCGCAA|-GATCACATTTTTGGTCCCGTTCACTGCTGGCT
	0	29%		CGGACTTTGGCCTCAGCCGCCGCAA|TGATCACATTTTTGGTCCCGTTCACTGCTGGCT
Cell cluster 81	0	62%	0.98	CGGACTTTGGCCTCAGCCGCCGCAA|TGATCACATTTTTGGTCCCGTTCACTGCTGGCT
	+1	19%		CGGACTTTGGCCTCAGCCGCCGCAA|nTGATCACATTTTTGGTCCCGTTCACTGCTGGCT
	-1	17%		CGGACTTTGGCCTCAGCCGCCGCAA|-GATCACATTTTTGGTCCCGTTCACTGCTGGCT

Table S3 (cont.): Contribution of INDELs in proliferating cell clusters with significant gene editing in the *CCR2* locus.

	INDEL	Contribution (%)	R Squared	Sequence
Cell cluster 82	0	91%	0.99	CGGACTTTGGCCTCAGCCGCCGCAA|TGATCACATTTTTGGTCCCGTTCACTGCTGGCT
	+1	5%		CGGACTTTGGCCTCAGCCGCCGCAA|nTGATCACATTTTTGGTCCCGTTCACTGCTGGCT
	-1	3%		CGGACTTTGGCCTCAGCCGCCGCAA|-GATCACATTTTTGGTCCCGTTCACTGCTGGCT
Cell cluster 83	0	85%	0.99	CGGACTTTGGCCTCAGCCGCCGCAA|TGATCACATTTTTGGTCCCGTTCACTGCTGGCT
	+1	12%		CGGACTTTGGCCTCAGCCGCCGCAA|nTGATCACATTTTTGGTCCCGTTCACTGCTGGCT
	-1	2%		CGGACTTTGGCCTCAGCCGCCGCAA|-GATCACATTTTTGGTCCCGTTCACTGCTGGCT
Cell cluster 90*	0	9%	0.36	CGGACTTTGGCCTCAGCCGCCGCAA|TGATCACATTTTTGGTCCCGTTCACTGCTGGCT
	+210			CGGACTTTGGCCTCAGCCGCCGCAA|Tn_{210}GATCACATTTTTGGTCCCGTTCACTGCTGGCT
Cell cluster 92	0	99%	1	CGGACTTTGGCCTCAGCCGCCGCAA|TGATCACATTTTTGGTCCCGTTCACTGCTGGCT
	+1	1%		CGGACTTTGGCCTCAGCCGCCGCAA|nTGATCACATTTTTGGTCCCGTTCACTGCTGGCT
Cell cluster 97	0	97%	0.99	CGGACTTTGGCCTCAGCCGCCGCAA|TGATCACATTTTTGGTCCCGTTCACTGCTGGCT
	-1	2%		CGGACTTTGGCCTCAGCCGCCGCAA|-GATCACATTTTTGGTCCCGTTCACTGCTGGCT
Cell cluster 98*	0	19%	0.43	CGGACTTTGGCCTCAGCCGCCGCAA|TGATCACATTTTTGGTCCCGTTCACTGCTGGCT
	+154			CGGACTTTGGCCTCAGCCGCCGCAA|n_{154}TGATCACATTTTTGGTCCCGTTCACTGCTGGCT
Cell cluster 99	-1	48%	0.97	CGGACTTTGGCCTCAGCCGCCGCAA|-GATCACATTTTTGGTCCCGTTCACTGCTGGCT
	-3	47%		CGGACTTTGGCCTCAGCCGCCGCAA|---TCACATTTTTGGTCCCGTTCACTGCTGGCT
	0	2%		CGGACTTTGGCCTCAGCCGCCGCAA|TGATCACATTTTTGGTCCCGTTCACTGCTGGCT
Cell cluster 100*	0	98%	0.98	CGGACTTTGGCCTCAGCCGCCGCAA|TGATCACATTTTTGGTCCCGTTCACTGCTGGCT
	+106			CGGACTTTGGCCTCAGCCGCCGCAA|n_{106}TGATCACATTTTTGGTCCCGTTCACTGCTGGCT

Table S4: Contribution of INDELs in white-to-yellowish parental callus tissue. Synthego's ICE analysis tool was used to determine the sequence profile.

	INDEL	Contribution (%)	R squared	Sequence
**WT**	**0**	**100%**	**1**	**CGGACTTTGGCCTCAGCCGCCGCAA|TGATCACATTTTTGGTCCCGTTCACTGCTGGCT**
Parental callus 1	0	100%	1	CGGACTTTGGCCTCAGCCGCCGCAA|TGATCACATTTTTGGTCCCGTTCACTGCTGGCT
Parental callus 2	0	100%	1	CGGACTTTGGCCTCAGCCGCCGCAA|TGATCACATTTTTGGTCCCGTTCACTGCTGGCT
Parental callus 3	0	100%	1	CGGACTTTGGCCTCAGCCGCCGCAA|TGATCACATTTTTGGTCCCGTTCACTGCTGGCT
Parental callus 4	0	100%	1	CGGACTTTGGCCTCAGCCGCCGCAA|TGATCACATTTTTGGTCCCGTTCACTGCTGGCT
Parental callus 5	0	100%	1	CGGACTTTGGCCTCAGCCGCCGCAA|TGATCACATTTTTGGTCCCGTTCACTGCTGGCT
Parental callus 6	0	100%	1	CGGACTTTGGCCTCAGCCGCCGCAA|TGATCACATTTTTGGTCCCGTTCACTGCTGGCT
Parental callus 7	0	100%	1	CGGACTTTGGCCTCAGCCGCCGCAA|TGATCACATTTTTGGTCCCGTTCACTGCTGGCT
Parental callus 8	0	100%	0.99	CGGACTTTGGCCTCAGCCGCCGCAA|TGATCACATTTTTGGTCCCGTTCACTGCTGGCT
Parental callus 9	0	100%	1	CGGACTTTGGCCTCAGCCGCCGCAA|TGATCACATTTTTGGTCCCGTTCACTGCTGGCT
Parental callus 10	0	100%	1	CGGACTTTGGCCTCAGCCGCCGCAA|TGATCACATTTTTGGTCCCGTTCACTGCTGGCT

Table S5: Predicted sequence of the *P. alba 18S* ribosomal RNA sequence (accession XR _004689093). Category III mutations were characterized by the insertion of *Populus* genomic DNA fragments at the Cas9 target site. In six independent lines (42.8, 43.5, 44.2, 45.3, 48.1, 84.7) a 76-bp insertion (underlined) was observed originating from the multicopy endogenous *18S* rRNA gene.

>XR_004689093.1 PREDICTED: *Populus alba* 18S ribosomal RNA (LOC118058089), rRNA

TACCTGGTTGATCCTGCCAGTAGTCATATGCTTGTCTCAAAGATTAAGCCATGCATGTGTA
AGTATGAACTAATTCAGACTGTGAAACTGCGAATGGCTCATTAAATCAGTTATAGTTTGT
TTGATGGTATTTGCTACTCGGATAACCGTAGTAATTCTAGAGCTAATACGTGCAACAAAC
CCCGACTTCTGGAAGGGACGCATTTATTAGATAAAAGGTCGACGCGGGCTCTGCCCGTTG
CTCTGATGATTCATGATAACTCGACGGATCGCACGGCCATCGTGCTGGCGACGCATCATT
CAAATTTCTGCCCTATCAACTTTCGATGGTAGGATAGAGGCCTACCATGGTGGTGACGGG
TGACGGAGAATTAGGGTTCGATTCCGGAGAGGGAGCCTGAGAAACGGCTACCACATCCA
AGGAAGGCAGCAGGCGCGCAAATTACCCAATCCTGACACGGGGAGGTAGTGACAATAAA
TAACAATACCGGGCTCTTCGAGTCTGGTAATTGGAATGAGTACAATCTAAATCCCTTAAC
GAGGATCCATTGGAGGGCAAGTCTGGTGCCAGCAGCCGCGGTAATTCCAGCTCCAATAG
CGTATATTTAAGTTGTTGCAGTTAAAAAGCTCGTAGTTGGACTTTGGGTTGGGTCGGCCG
GTCCGCCTCAGGTGTGCACCGGTCGCCTCGTCCCTTCTACCGGCGATGCGCTCCTGGCCTT
AAACTGGCCGGGTCGTGCCTCCGGTGCTGTTACTTTGAAGAAATTAGAGTGCTCAAAGCA
AGCCTACGCTCTGGATACATTAGCATGGGATAACATCATAGGATTTCGATCCTATTGTGTT
GGCCTTCGGGATCGGAGTAATGATTAACAGGGACAGTCGGGGGCATTCGTATTTCATAGT
CAGAGGTGAAATTCTTGGATTTATGAAAGACGAACAACTGCGAAAGCATTTGCCAAGGA
TGTTTTTCATTAATCAAGAACGAAAGTTGGGGGCTCGAAGACGATCAGATACCGTCCTAG
TCTCAACCATAAACGATGCCGACCAGGGATTGGCGGATGTTGCTTCTAGGACTCCGCCAG
CACCTTATGAGAAATCAAAGTTTTTGGGTTCTGGGGGGAGTATGGTCGCAAGGCTGAAAC
TTAAAGGAATTGACGGAAGGGCACCACCAGGAGTGGAGCCTGCGGCTTAATTTGACTCA
ACACGGGGAAACTTACCAGGTCCAGACATAGTAAGGATTGACAGACTGAGAGCTCTTTCT
TGATTCTATGGGTGGTGGTGCATGGCCGTTCTTAGTTGGTGGAGCGATTTGTCTGGTTAAT
TCCGTTAACGAACGAGACCTCAGCCTGCTAACTAGCTATGCGGAGGTGACCCTCCGCGGC
CAGCTTCTTAGAGGGACTATGGCCTTCCAGGCCAAGGAAGTTTGAGGCAATAACAGGTCT
GTGATGCCCTTAGATGTTCTGGGCCGCACGCGCGCTACACTGATGTATTCAACGAGTCTA
TAGCCTTGGCCGACAGGCCCGGGTAATCTTTGAAATTTCATCGTGATGGGGATAGATCAT
TGCAATTGTTGGTCTTCAACGAGGAATTCCTAGTAAGCGCGAGTCATCAGCTCGCGTTGA
CTACGTCCTGCCCTTTGTACACACCGCCCGTCGCTCCTACCGATTGAATGGTCCGGTGAAG
TGTTCGGATCGCGCGACGTGGGCGGTTCGCCGCCGGCGACGTCGCGAGAAGTCCACTGA
ACCTTATCATTTAGAGGAAGGAGAAGTCGTAACAAGGTTTCCGTAGGTGAAACCTGCGG
AACGATCATTG

Supplementary references

De Meester, B., Madariaga Calderón, B., De Vries, L., Pollier, J., Goeminne, G., Van Doorsselaere, J., et al. (2020). Tailoring poplar lignin without yield penalty by combining a null and haploinsufficient *CINNAMOYL-CoA REDUCTASE2* allele. *Nature Communications* 11**,** 5020.

Leplé, J.-C., Dauwe, R., Morreel, K., Storme, V., Lapierre, C., Pollet, B., et al. (2007). Downregulation of *cinnamoyl-coenzyme A reductase* in poplar: multiple-level phenotyping reveals effects on cell wall polymer metabolism and structure. *Plant Cell* 19**,** 3669-3691. doi:10.1105/tpc.107.054148

Leplé, J. C., Brasileiro, A. C. M., Michel, M. F., Delmotte, F., and Jouanin, L. (1992). Transgenic poplars: expression of chimeric genes using four different constructs. *Plant Cell Rep* 11**,** 137-141.

Ralph, J., Kim, H., Lu, F., Grabber, J. H., Leplé, J.-C., Berrio-Sierra, J., et al. (2008). Identification of the structure and origin of a thioacidolysis marker compound for ferulic acid incorporation into angiosperm lignins (and an indicator for cinnamoyl CoA reductase deficiency). *Plant J.* 53**,** 368-379. doi:10.1111/j.1365-313X.2007.03345.x
